# Development and validation of a versatile foundation model for cine cardiac magnetic resonance image analysis

**DOI:** 10.1038/s43856-026-01636-0

**Published:** 2026-05-13

**Authors:** Yunguan Fu, Wenjia Bai, Weixi Yi, Charlotte Manisty, Anish N. Bhuva, Thomas A. Treibel, James C. Moon, Matthew J. Clarkson, Rhodri Huw Davies, Yipeng Hu

**Affiliations:** 1 UCL Hawkes Institute, University College London, London, UK; 2 InstaDeep, London, UK; 3 Imperial College London, London, UK; 4 Institute of Cardiovascular Sciences, University College London, London, UK; 5 Barts Heart Centre, Barts Health NHS Trust, London, UK

**Keywords:** Cardiovascular biology, Cardiovascular diseases

## Abstract

**Background:**

Cardiac magnetic resonance imaging is central to cardiovascular diagnosis and management, yet extracting key clinical measurements remains time-consuming, subjective, and of limited reproducibility. Current deep learning methods often require a separate model trained from scratch for each task, and generating sufficient labelled training data demands substantial clinical expertise.

**Methods:**

We developed CineMA, a multi-view conv-transformer masked autoencoder foundation model, pre-trained on 15 million cine cardiac magnetic resonance images from 74,916 studies. The model was fine-tuned and evaluated on eight independent datasets for segmentation, landmark localisation, disease diagnosis, and prognostication, representing the largest such benchmark to date. Performance was compared against convolutional neural network baselines, including nnUNet.

**Results:**

Here we show, without dataset-specific hyperparameter tuning, CineMA approaches nnUNet performance in ventricle segmentation and ejection fraction estimation while achieving higher consistency across repeated scans. CineMA surpasses convolutional baselines in cardiovascular disease detection with notably improved specificity, and matches their performance in long-axis function measurement. Beyond cardiac diseases, CineMA shows potential for predicting systemic conditions and survival outcomes, with comparable performance across demographic subgroups.

**Conclusions:**

CineMA demonstrates accuracy, learning efficiency, adaptability, and fairness across diverse cardiac image analysis tasks, offering a strong alternative to task-specific model training for automated cardiac image analysis.

## Plain Language Summary

Cardiac magnetic resonance imaging produces detailed images of the heart that help doctors diagnose and monitor heart conditions. However, analysing these images is time-consuming and requires expert interpretation, which can vary between clinicians. We developed CineMA, an artificial intelligence model trained on over 15 million heart images from nearly 75,000 studies. Unlike common approaches requiring separate models for each task, CineMA can perform multiple analysis tasks including measuring heart function, detecting diseases, and predicting patient outcomes. Testing on eight independent datasets showed that CineMA performs comparably to specialised models while being more consistent across different populations. Beyond heart disease, CineMA also showed potential for predicting conditions such as hypertension and diabetes. All code and models are freely available, enabling researchers worldwide to build upon this work.

## Abstract (web summary)

Fu et al. develop CineMA, a foundation model pre-trained on 15 million cine cardiac MRI images. CineMA performs competitively in segmentation, disease detection, landmark localisation and prognostication across eight independent datasets, offering a versatile alternative to task-specific models.

## Introduction

Despite major advances over recent decades, cardiovascular diseases remain the leading cause of mortality worldwide, accounting for over 19.8 million deaths annually^1^. Many diagnostic decisions are driven by imaging assessment of cardiac structure and function. Due to its excellent soft tissue contrast, non-invasiveness, and absence of ionising radiation, cardiovascular magnetic resonance (CMR) is now a standard part of many clinical pathways as reflected in many international guidelines^2–4^. Cardiac magnetic resonance images encompass multiple sequences, including cine imaging for functional assessment, late gadolinium enhancement (LGE) for scar detection, and T1/T2 mapping for tissue characterisation. Among these, cine imaging is the most widely acquired sequence and enables quantification of cardiac size (e.g. cavity volume), myocardial mass, global systolic function (e.g. left ventricular ejection fraction [LVEF]) and long-axis function (e.g. mitral annular plane systolic excursion [MAPSE] and global longitudinal shortening [GLS]). CMR is established as the gold standard for estimating these measures and providing both diagnostic and prognostic value^5^. However, interpreting CMR images and deriving biomarkers for diagnosis, monitoring and prognostication remain labour-intensive tasks that require considerable effort by experienced clinicians. In addition, the annotation process is inherently subjective, contributing to substantial intra- and inter-observer variability^6^, which can affect clinical decision-making and compromise consistency in patient care. This variability is compounded when CMR measurements are compared with echocardiography, which remains the frontline imaging modality in many settings. With the advancement of deep learning, automated image interpretation has become feasible, helping to alleviate clinician workload, standardise quantitative cardiac assessment, and improve clinical workflow efficiency^6–13^.

One particular advancement in deep learning is foundation models^14^, models trained on large datasets and adaptable to diverse downstream tasks. Such models hold great potential for CMR imaging analyses, which involve diverse tasks from segmentation, landmark localisation, to diagnosis and prognostication. MedSAM^15^ is one such foundation model, developed for general 2D medical image segmentation. However, it is not fully automated, requiring manually provided bounding box prompts to perform segmentation. Moreover, it cannot perform other CMR analysis tasks. Sun et al.^16^ developed a foundation model for correcting motion, enhancing resolution and denoising brain MR images. The enhanced images yielded improved performance in downstream tasks, including tissue segmentation, registration and diagnosis. Several foundation models tailored specifically to CMR have recently been proposed^17–19^. Shad et al.^17^ used 14,073 cardiac MRI scans to align vision and text encoders, and then fine-tuned the vision encoder for LVEF prediction and disease detection on the ACDC dataset^7^. Jacob et al.^18^ introduced a 2D model trained on 27,524 CMR studies using the DINO pre-training framework^20^, and evaluated it for disease detection, ventricle segmentation and landmark localisation tasks across multiple datasets, including ACDC, Kaggle^10^ and EMIDEC^12^. More recently, Zhang et al.^19^ proposed a multi-modal foundation model by training a multi-view masked autoencoder^21^ on 42,000 CMR studies and aligning image embeddings with tabular clinical features using contrastive learning. Their model was evaluated on UK Biobank (UKB) data across multiple tasks, including ejection fraction (EF) estimation, disease classification and segmentation. These studies provide the first evidence that pre-training on large cine CMR datasets can improve downstream performance. However, their evaluations were limited in several aspects, such as the absence of convolutional neural network baselines^19^, the omission of clinically relevant metrics, and the lack of validation on external datasets^19^. While the reported results are promising, the proposed models did not consistently match strong convolutional baselines.

Here we present CineMA (cine CMR masked autoencoder), a cine CMR foundation model using the self-supervised masked autoencoder framework^21^, trained on 74,916 cine CMR studies comprising over 15 million images from the UKB. We hypothesise that large-scale pre-training yields transferable representations for downstream tasks. We benchmark fine-tuned variants of CineMA against convolutional neural networks (CNNs), including nnUNet^22^, across a range of clinically relevant tasks. These tasks represent key components of cardiac image interpretation in routine practice, including segmentation, landmark localisation, diagnosis and prognostication. CineMA demonstrates segmentation accuracy competitive with nnUNet and higher EF estimation consistency in low-data settings with population shifts. CineMA consistently outperforms CNNs in cardiac disease diagnosis, achieving notably improved specificity and suggesting potential for triage and stratification. CineMA matches CNNs in long-axis function estimation, demonstrating adaptability. In exploratory analyses, CineMA shows potential for predicting non-cardiac conditions (diabetes, hypertension, cancer) and long-term survival, with few performance disparities across demographic subgroups. These analyses highlight the broader applicability and trustworthiness of CineMA in real-world populations and support the use of the proposed foundation model as an alternative to task-specific training for future cardiac imaging applications. All code for pre-training, fine-tuning and inference, along with pre-trained and fine-tuned models, is publicly available at https://github.com/mathpluscode/CineMA, enabling reproducibility and direct application of the foundation model.

## Methods

### Datasets

We used nine datasets spanning multiple institutions, scanner vendors and countries (Table 1): the UKB^23^, the automated cardiac diagnosis challenge (ACDC)^7^, the multi-centre, multi-vendor and multi-disease cardiac image segmentation challenge (M&Ms)^8^, M&Ms2^9^, the Kaggle dataset^10^, the Rescan dataset^6^, the Landmark dataset^11^, the EMIDEC dataset^12^ and the MyoPS2020 dataset^13^. Together, these datasets cover cine, LGE, T2-weighted and balanced steady-state free precession (bSSFP) CMR sequences in both short-axis and long-axis views.

**Table 1:** Datasets used in pre-training and downstream tasks

Dataset	Modality	Views	*n*	Tasks	Scanner	Country
UKB^23^	Cine	SAX; LAX 2C/3C/4C	74,916	Pre-training; diabetes, hypertension, cancer; 1-/2-/3-year survival.	1.5T Siemens Aera.	UK
ACDC^7^	Cine	SAX	150	Ventricle segmentation; EF and BMI regression; disease classification.	1.5T Siemens Aera, 3T Siemens Trio Tim.	France
M&Ms^8^	Cine	SAX	375	Ventricle segmentation; EF and age regression; disease and sex classification.	1.5T Siemens Avanto, 1.5T Philips Achieva, 1.5T GE Signa Excite, 1.5T Canon Vantage Orian, 3T Siemens Skyra.	Spain, Canada, Germany
M&Ms2^9^	Cine	SAX; LAX 4C	360	Ventricle segmentation; EF regression; disease and vendor classification.	1.5T Philips Achieva, 1.5T GE Signa Excite/HDxt/explorer, 1.5T Siemens Avanto (Fit), 1.5T Siemens Symphony (Tim), 3T GE Signa HDxt, 3T Siemens Trio Tim.	Spain
Kaggle^10^	Cine	SAX	397	EF regression.	N/A	US
Rescan^6^	Cine	SAX	206	EF regression.	1.5T, 3T Siemens, Philips Aera, Achieva, Avanto.	UK
Landmark^11^	Cine	LAX 2C/4C	1453	Landmark localisation; long-axis function regression.	1.5T Siemens Aera, 3T Siemens Magnetom Prisma	UK
EMIDEC^12^	LGE	SAX	100	Ventricle segmentation.	1.5T/3T Siemens	France
MyoPS2020^13^	LGE, T2, bSSFP	SAX	45	Scar segmentation.	1.5T Philips Achieva	China

*n* is the number of subjects or labelled samples used in this study for each dataset. Each dataset includes different views and may contain a different number of frames.

*SAX* short-axis, *LAX* long-axis, *nC*, n-chamber, *EF* ejection fraction, *BMI* body mass index, *LGE* late gadolinium enhancement, *bSSFP* balanced steady-state free precession.

#### Pre-training

We curated 74,916 unlabelled cine CMR studies from UKB (up to 6 November 2023) using data fields 20208 (LAX heart images) and 20209 (SAX heart images). The LAX images included two-chamber, three-chamber and four-chamber views. Each study contained 50 images. Both Instance 2 (first imaging visit) and Instance 3 (first repeat imaging visit) were used.

#### Ventricle and myocardium segmentation

We collected 885 SAX cine CMR scans and corresponding segmentation masks of the ventricles and myocardium from ACDC, M&Ms and M&Ms2. M&Ms2 additionally contains LAX 4C views and corresponding segmentation masks. For each subject, only the end-diastolic (ED) and end-systolic (ES) frames were used for training and evaluation. Ventricular and myocardial volumes were calculated as the product of the number of labelled voxels and the voxel volume. The dataset includes both healthy subjects and those with cardiovascular disease. For M&Ms and M&Ms2, we used the official challenge train/validation/test splits (M&Ms: 175/40/160; M&Ms2: 160/40/160 subjects). For ACDC, we used the official 100/50 train/test split and further held out two subjects per pathology (10 subjects in total) from the training set as a validation set, yielding a 90/10/50 split. The same splits were reused for the downstream EF estimation and disease classification tasks on these datasets.

#### Ejection fraction estimation

For ejection fraction prediction, we derived ventricular and myocardial volumes from the 885 segmentation masks in the ACDC, M&Ms and M&Ms2 datasets. EF was then calculated from the end-diastolic volume (EDV) and end-systolic volume (ESV) using the formula:

$${\mbox{EF}}=\frac{{\mbox{EDV}}-{\mbox{ESV}}}{{\mbox{EDV}}}\times 100 \% .$$

To assess generalisability, we additionally included 397 Kaggle cases and 206 short-axis cine CMR scans from the Rescan dataset, together with their LVEF labels. These scans were not used for model training and serve as an external evaluation set. This combined external dataset represents a population-shifted distribution, with a higher average LVEF compared to the other datasets. Since the Rescan scans are paired, corresponding to acquisitions at two time points that are within one week in 96% of cases, their LVEF measurements were averaged to create a shared reference label for the 102 complete repeated-scan pairs used in CV analysis.

#### Cardiovascular disease diagnosis

For disease classification, we used the ED and ES frames from the ACDC, M&Ms and M&Ms2 datasets, each of which contains a distinct set of diagnostic categories (Table 2). To create a unified binary disease detection task, we pooled all disease types into a single 'disease present' class. This binary detection dataset comprised all 885 SAX samples. Binary detection performance was evaluated on the combined held-out SAX test splits from ACDC, M&Ms and M&Ms2 after excluding samples whose labels were absent from the corresponding training split (*n* = 263).

**Table 2:** Cardiovascular disease distribution in ACDC, M&Ms and M&Ms2 datasets

Class	ACDC				M&Ms				M&Ms2			
	Train	Val	Test	*n*	Train	Val	Test	*n*	Train	Val	Test	*n*
NOR	18	2	10	30	38	9	31	78	40	5	30	75
HCM	18	2	10	30	49	9	23	81	30	5	23	58
DCM	18	2	10	30	54	8	35	97				
ARV	18	2	10	30	8	1	6	15		5	24	29
MINF	18	2	10	30								
ARR									20	5	10	35
FALL									17	5	10	32
CIA									20	5	10	35
DLV									29	3	25	57
TRI										5	25	30
AHS						1		1				
HHD					1	4	10	15				
IHD							3	3				
LVNC							2	2				
Other						1	24	25				

Samples whose label does not exist in training were excluded from test set in this study.

*NOR* normal, *HCM* hypertrophic cardiomyopathy, *DCM* dilated cardiomyopathy, *ARV* arrhythmogenic right ventricular cardiomyopathy, *MINF* myocardial infarction, *ARR* arrhythmia, *FALL* tetralogy of Fallot, *CIA* congenital interatrial communication, *DLV* dilated left ventricle, *AHS* athlete’s heart syndrome, *HHD* hypertensive heart disease, *IHD* ischemic heart disease, *LVNC* left ventricular non-compaction, *TRI* tricuspid abnormality.

#### Long-axis function estimation

The processed Landmark dataset comprised 1453 unique subjects with annotated LAX 2C and/or 4C images at ED and ES phases, and was used to estimate long-axis function. Subjects were randomly split 60:20:20 into training, validation and test sets, with the same split shared across the LAX 2C and LAX 4C views. For each subject, we calculated mitral annular plane systolic excursion (MAPSE) as the average displacement of the mitral valve landmarks from ED to ES (Eq. 1):

1 $${\mbox{MAPSE}}=\frac{1}{2}(\parallel {{{{\bf{p}}}}}_{1}^{{\mbox{ED}}}-{{{{\bf{p}}}}}_{1}^{{\mbox{ES}}}\parallel +\parallel {{{{\bf{p}}}}}_{2}^{{\mbox{ED}}}-{{{{\bf{p}}}}}_{2}^{{\mbox{ES}}}\parallel ),$$

where ${{\bf{p}}}_{1}^{{\mbox{ED}}},{{\bf{p}}}_{2}^{{\mbox{ED}}},{{\bf{p}}}_{1}^{{\mbox{ES}}},{{\bf{p}}}_{2}^{{\mbox{ES}}}\in {{\mathbb{R}}}^{2}$ are the 2D coordinates of the mitral annular landmarks at ED and ES, and ∥⋅∥ denotes the Euclidean norm. For each LAX image, we estimated the ventricular length as the distance between the midpoint of the mitral annular landmarks and the apical landmark (Eq. 2):

2 $${\mbox{LV\,Length}}=\parallel \frac{{{{{\bf{p}}}}}_{1}+{{{{\bf{p}}}}}_{2}}{2}-{{{{\bf{p}}}}}_{{\mbox{apex}}}\parallel ,$$

where ${{{{\bf{p}}}}}_{{\mbox{apex}}}\in {{\mathbb{R}}}^{2}$ denotes the 2D coordinates of the apical landmark. Finally, GLS was estimated in a form analogous to EF (Eq. 3):

3 $${{\mbox{GLS}}}=\frac{{{\mbox{ED}}} \, {{\mbox{LV}}} \, {{\mbox{Length}}}\,-\,{{\mbox{ES}}} \, {{\mbox{LV}}} \, {{\mbox{Length}}}} {{{\mbox{ED}}} \, {{\mbox{LV}}} \, {{\mbox{Length}}}} \times 100 \% .$$

Additionally, for each image, a heatmap was generated per landmark using a Gaussian kernel with *σ* = 3 (Eq. 4):

4 $${{\mbox{heatmap}}}({{{\bf{p}}}};{{{{\bf{p}}}}}_{i})=\exp (-\frac{\parallel {{{\bf{p}}}}-{{{{\bf{p}}}}}_{i}{\parallel }^{2}}{2{\sigma }^{2}})$$

where ${{{\bf{p}}}}\in {{\mathbb{R}}}^{2}$ denotes a pixel location and ${{{{\bf{p}}}}}_{i}\in {{\mathbb{R}}}^{2}$ is the ground truth coordinate of the *i*-th landmark, with *i* ∈ {1, 2, apex}. The three landmark heatmaps form a three-class soft segmentation mask, and Dice loss combined with cross-entropy loss on this mask was used for training.

#### Systemic disease and survival outcome predictions

We used UKB for predicting diabetes, cancer, hypertension and survival status. Hypertension, cancer and diabetes are prevalent disease predictions, as the labels reflect whether each condition had ever been diagnosed at the time of data retrieval, rather than incident events occurring after imaging. The labels of diabetes, hypertension and cancer were derived from data fields 2443 (diabetes diagnosed by doctor), 2966 (age high blood pressure diagnosed), and 40008 (age at cancer diagnosis), respectively. As UKB regularly updates the metadata, for individuals diagnosed with hypertension or cancer after the image acquisition, i.e. the age at diagnosis is larger than the age at acquisition, the labels were set to negative. One-year, 2-year and 3-year survival labels were derived from data field 40007 (age at death), where a positive label indicates death within the respective time window after imaging. Since the data was retrieved in 2023, participants were included only if sufficient follow-up time had elapsed (e.g. imaging visit in 2022 or earlier for 1-year survival, 2020 or earlier for 3-year survival); participants with insufficient follow-up were excluded. If the age at death was not recorded, the participant was assumed to have survived. Sex and ethnic group information were obtained from data fields 31 and 21000 (ethnic background), respectively. The LVEF labels were provided by data field 22420 (LV ejection fraction), which was automatically derived from heart MRI data (inlineVF) without any expert quality control. A comparison of the demographic distribution to other datasets is illustrated in Supplementary Fig. 1.

For classification tasks on UKB, datasets were split temporally into training (records before 2019), validation (2019) and test (after 2019) sets (Table 3) to simulate a prospective study. To prepare input data, fine-tuned CineMA segmentation and landmark models were first applied to derive ventricular and myocardial volumes from SAX views, and functional metrics (MAPSE and GLS) from LAX views. End-diastolic and end-systolic frames were identified as those with maximum and minimum left ventricular volumes, respectively. Only these two frames were used as input for fine-tuning CineMA classification models. For GLM and XGBoost baselines, summary statistics (mean, standard deviation, minimum and maximum) of the derived morphometric and functional metrics across all frames were used as features.

**Table 3:** Distribution of systemic diseases and survival labels in UK Biobank datasets

Label	Split	*N*	Y	Y%	N/A
Hypertension	Train	21,585	8551	28.37	
	Val	11,027	4499	28.98	
	Test	20,984	9402	30.94	
Cancer	Train	25,931	4205	13.95	
	Val	13,102	2424	15.61	
	Test	25,033	5353	17.62	
Diabetes	Train	29,130	1006	3.34	
	Val	15,059	467	3.01	
	Test	29,559	827	2.72	
1-year survival	Train	30,096	34	0.11	6
	Val	15,504	18	0.12	4
	Test	17,412	18	0.10	12,956
2-year survival	Train	30,002	128	0.42	6
	Val	15,463	59	0.38	4
	Test	6248	32	0.51	24,106
3-year survival	Train	29,902	228	0.76	6
	Val	15,405	117	0.75	4
	Test	2734	33	1.19	27,619

Values show the number of positive and negative samples in training, validation and test sets.

#### Scar segmentation

We additionally used the 100 labelled DE-MRI images from the EMIDEC training set and 45 studies from MyoPS2020, comprising LGE, T2-weighted and bSSFP sequences. The full EMIDEC dataset contains 150 images, but labels are available only for the 100 images in the training partition; these were randomly divided into training, validation and test sets in a 60:20:20 ratio, and performance is reported on the held-out test set. For MyoPS2020, 25 studies were allocated to model development, with 20 used for training and 5 for validation, and performance is reported on the independent test set of 20 studies. Both datasets were acquired in the short-axis view but exhibit image-intensity distributions distinct from those of standard cine CMR. Both datasets provide segmentation labels for the ventricular cavity, myocardium and myocardial scars.

### Data preprocessing

All image resolutions were standardised to 1 × 1 × 10 mm for SAX and 1 × 1 mm for LAX views. All images were then centre-cropped or padded to a fixed spatial size of 192 × 192 for SAX and 256 × 256 for LAX. Centre cropping was performed either at the intersection of SAX, LAX 2C and LAX 4C views when available or at the anatomical centre of the left ventricle.

### CineMA architecture and implementation

CineMA adopts a masked autoencoder architecture^21^ that processes four views: two-chamber, three-chamber and four-chamber LAX views, along with SAX views. The LAX views are two-dimensional with size (256, 256), while the SAX views are three-dimensional with size (192, 192 and 16). Each view is first split into patches of size (16, 16), with the SAX views using a depth-wise patch size of 1. A fixed proportion (75%) of patches is randomly masked by setting them to zero. The input images are then encoded using convolutional downsampling as in MCMAE^24^, reducing the spatial dimensions by a factor of 8×. The downsampled features are split into non-overlapping (2, 2) patches and embedded into feature tokens. Each masked patch corresponds to a single feature token, allowing the tokens to be categorised into two groups: visible tokens (from unmasked patches) and masked tokens (to be predicted). Following MultiMAE^25^, the visible tokens from all views are concatenated and passed through a shared vision transformer encoder. The encoded representations are then combined with the masked tokens and passed through a shared transformer decoder to reconstruct the masked patches.

In this study, CineMA adopted the 'base' configuration from He et al.^21^, resulting in a model with 126 million parameters. It was pre-trained for 800 epochs on 74,916 cine CMR studies from the UKB^23^, using LAX 2C, 3C, 4C and SAX views. For each study, a single image was randomly selected from the available 50 frames. Random gamma augmentation was applied, followed by independent random affine transformations (including rotation, zooming, shearing and shifting) to each view. The model was trained with a batch size of 128. The learning rate schedule involved a linear warm-up over the first 10 epochs from 0 to 0.001, followed by a cosine decay to 10^−6^. Training used a mean squared error loss on masked patches and the AdamW optimiser with betas (0.9, 0.95). Gradients were clipped with a maximum norm of 5.0. The final checkpoint was used for all downstream tasks.

### Baseline models

For dense prediction tasks such as segmentation and heatmap-based landmark localisation, five-layer UNet models with residual connections were employed. These models used channel widths of 32, 64, 128, 256 and 512 across successive layers. For tabular labels, such as EF estimation and disease classification, ResNet-50^26^ was used as the baseline model. 2D and 3D models were used for LAX and SAX images, respectively. All CNNs were trained using the same procedures as those used for fine-tuning CineMA. nnUNet^22^ was also included in segmentation benchmarks using the latest nnUNetResEncUNetMPlans configuration, whereas hyperparameter optimisation was not performed for fine-tuned CineMA.

With convolutional layers in CineMA, the effective patch size before the transformer is 16 × 16 for SAX slices. A transformer-only model is constructed by removing the convolutional layers and using a patch size of 16 × 16. Reducing the patch size to 4 × 4, corresponding to the input patch size used in the convolutional setting, would increase the number of tokens 16-fold, slowing training by more than 10 times and rendering both pre-training and downstream fine-tuning infeasible.

### Downstream training and evaluation

In downstream task fine-tuning, we retained only the encoder branches corresponding to the available views and discarded all decoder layers. Additional layers were added with randomly initialised weights depending on the type of label. A UNetR-style architecture^27^ was employed for segmentation masks and landmark location heatmaps. For tabular labels, such as disease classification or EF values, linear prediction layers were added. During fine-tuning, a weight decay of 0.05 was applied to the transformer layers. All model weights were learnable, except in systemic disease and survival outcome prediction tasks, where only the last block of the pre-trained transformer was learnable. Data augmentation techniques included random gamma adjustment, random scaling and random affine transformations for all tasks. Label smoothing was applied during classification training, and random image dropout was used during segmentation training. A linear warm-up followed by cosine decay learning rate schedule was used, and early stopping was employed based on task-specific validation metrics. For each task, all models were trained using the same protocols with identical training, validation and test splits to ensure fair comparisons. For all models except nnUNet, the same set of hyperparameters was used without exhaustive search (Supplementary Table 1).

Segmentation performance was assessed using the Dice score and 95th percentile Hausdorff distance. Disease classification tasks were evaluated using the area under the receiver operating characteristic curve (AUROC). For systemic disease and survival prediction tasks, the area under the precision-recall curve (AUPRC) was additionally reported due to class imbalance. A random classifier would achieve an AUROC of 0.5 and an AUPRC equal to the class prevalence. For disease detection, sensitivity and specificity were reported. Labels with continuous values such as volumes, EF, MAPSE and GLS were evaluated using mean absolute error. To evaluate model consistency across repeated acquisitions in the Rescan dataset, the coefficient of variation was calculated. Landmark localisation accuracy was evaluated using Euclidean distance. For each task, we trained the model three times using different random seeds, either on the full dataset or on a randomly sampled subset. When subsets were used, the training samples varied across seeds, while the validation set remained fixed. Metrics were calculated per trained model using predictions averaged across random seeds.

### Computational resources

The pre-training of CineMA was completed in 18 days using 8 NVIDIA RTX A6000 GPUs on the University College London Computer Science HPC cluster. Downstream training and fine-tuning were conducted on both the University College London Computer Science HPC cluster and the Isambard-AI cluster^28^. The duration of downstream training ranged from several hours to a maximum of 1 day, depending on dataset size and GPU availability.

### Statistics and reproducibility

Statistical significance of pairwise model comparisons was assessed using a two-sided Wilcoxon signed-rank test for Dice scores, ejection fraction errors, landmark distances, MAPSE and GLS. Significance levels are denoted *** for *p* < 0.001, ** for *p* < 0.01 and * for *p* < 0.05. No correction for multiple comparisons was applied. This study benchmarks model performance rather than testing a pre-specified clinical hypothesis. Reported metrics together with their mean ± standard deviation serve as direct, interpretable effect sizes, and *p*-values are reported as significance thresholds only.

### Ethics

This study used exclusively publicly available, de-identified datasets that have been previously published and are accessible under their respective data-sharing agreements. No new human data were collected, and no interventions were performed. Ethical approval was therefore not required.

## Results

### Model development

**Fig. 1:**
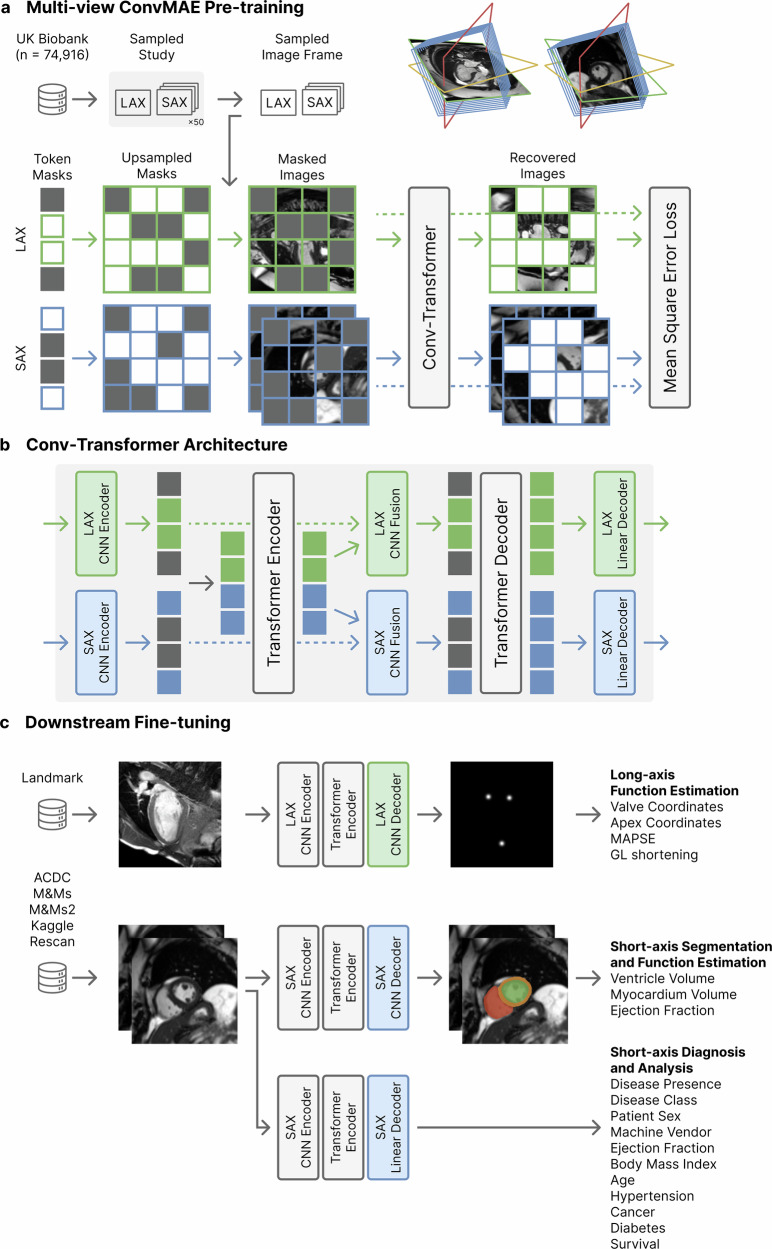
CineMA is a multi-view conv-transformer masked autoencoder. **a** CineMA was pre-trained on 74,916 cine cardiac MR studies. One image frame is sampled from a randomly selected study. Views are randomly masked and reconstructed using conv-transformer. Mean squared error loss is computed per view and then averaged. All three LAX views (two-chamber, three-chamber and four-chamber) were used, but only the two-chamber view is illustrated for simplicity. **b** Conv-transformer architecture. Each masked view is independently encoded by convolutional neural networks (CNNs). Visible patch tokens are concatenated and passed to a transformer encoder. After multi-scale fusion, visible and masked tokens are decoded by a transformer decoder to reconstruct each view. **c** CineMA was fine-tuned separately on multiple datasets to perform diverse clinically-relevant tasks. During fine-tuning, pre-trained decoders were discarded and replaced by task-specific heads. SAX short-axis, LAX long-axis, MAPSE mitral annular plane systolic excursion, GLS global longitudinal shortening, UKB UK Biobank.

Following Zhou et al.^29^, we adapted the MAE framework^21^ for model pre-training by reconstructing images from masked inputs. To support multiple views of cine CMR, including long-axis (LAX) and short-axis (SAX) views with a single unified backbone model, we adopted the MultiMAE architecture^25^, in which each view is encoded and decoded independently, before and after a shared transformer encoder. Following MCMAE^24^, we incorporated convolutional layers into the encoding and decoding stages to capture high-resolution local features while effectively reducing the token count. This hybrid approach provides a performance advantage (Supplementary Table 2) over purely transformer-based architectures with larger patch sizes and substantially accelerates training compared to transformers with small patch sizes. The proposed multi-view conv-transformer masked autoencoder model is named CineMA, short for cine CMR masked autoencoder (Fig. 1). CineMA was pre-trained on 74,916 cine CMR studies from UKB, comprising more than 15 million images and evaluated on eight downstream datasets (Table 1). During fine-tuning, encoder branches corresponding to unavailable views and decoder layers were removed. Task-specific heads were added depending on the downstream labels: UNetR-style architectures^27^ for segmentation masks, and linear prediction layers for tabular labels. We compared the performance of fine-tuned CineMA (denoted CineMA^FineTune^) against two baselines: (1) a model with the same architecture as CineMA^FineTune^ but trained from random initialisation (denoted CineMA^RandInit^), and (2) a convolutional neural network trained from random initialisation, UNet for segmentation labels (denoted UNet^RandInit^) and ResNet-50^26^ (denoted ResNet^RandInit^) for tabular labels. For segmentation tasks, we also compared against nnUNet^22^. Furthermore, on segmentation tasks, we compared the proposed conv-transformer architecture with a transformer-only model (Supplementary Table 2) and observed a drop in mean Dice score of 3.69–4.43% across all datasets (ACDC, M&Ms and M&Ms2). We therefore excluded transformer-only models from further comparisons.

### Ventricle and myocardium segmentation and ejection fraction prediction

We first evaluated CineMA by fine-tuning it for ventricular and myocardial segmentation on three independent short-axis cine CMR datasets (ACDC^7^, M&Ms^8^ and M&Ms2^9^). These cohorts represent a domain shift from the UKB pre-training data, encompassing diverse demographic distributions (Supplementary Fig. 1), heterogeneous imaging protocols (Table 1), and a wide spectrum of cardiovascular pathologies (Table 2). Consequently, they provide an important benchmark for assessing the model’s generalisability to clinical populations and imaging parameters not represented during pre-training. On the aggregated ACDC, M&Ms and M&Ms2 test sets, CineMA^FineTune^ achieved a mean Dice score of 88.72%, a substantial improvement over UNet^RandInit^ and CineMA^RandInit^. Furthermore, without extensive hyperparameter tuning, it achieved performance competitive with the nnUNet framework (89.24%), narrowing the gap to 0.52 percentage points. On the ACDC benchmark, Dice scores reached over 91.07% for the right ventricle (RV), 87.94% for the myocardium (MYO), and 93.63% for the left ventricle (LV), outperforming the previously reported scores of 90.7%, 87.9% and 93.3%, respectively, from Jacob et al.^18^ (Supplementary Table 3). CineMA^FineTune^ also produced the lowest Hausdorff distances (Supplementary Table 4), reflecting superior boundary delineation and anatomical consistency (Supplementary Fig. 2). The gains in segmentation accuracy translated into more precise volume estimation, with an average mean absolute error of 9.07 ml, comparable to nnUNet (8.98 ml) (Supplementary Table 5). When comparing end-diastolic and end-systolic volumes, CineMA^FineTune^ produced accurate ejection fraction estimates, with errors of 3.55% for LVEF and 5.56% for RVEF, comparable to nnUNet (3.64 and 5.17%) (Supplementary Table 6). These results highlight CineMA’s ability to deliver reliable ejection fraction estimates through accurate segmentation, establishing CineMA as a viable alternative to nnUNet for automated and reproducible cardiac function assessment in clinical practice.

### Cross-population LVEF prediction

**Fig. 2:**
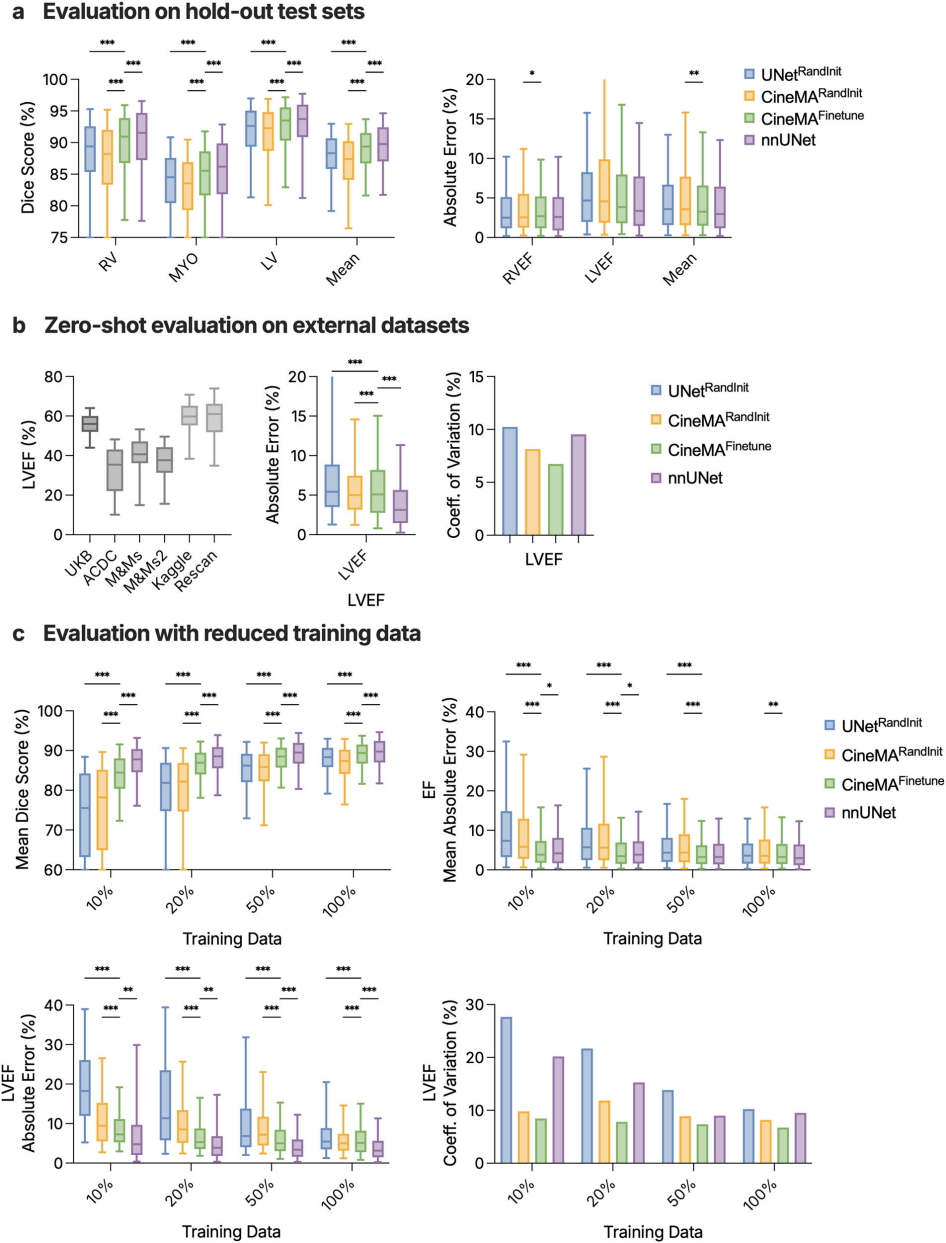
Segmentation model evaluation. **a** Models were compared on the combined test sets from ACDC, M&Ms and M&Ms2 (*n* = 682, with end-diastolic and end-systolic images as two separate samples) for Dice scores (left) and EF (right). Fine-tuned CineMA achieved performance comparable to nnUNet and outperformed other baselines. **b** Left: the pre-training dataset (UKB), fine-tuning datasets (ACDC, M&Ms and M&Ms2) and external datasets (Kaggle and Rescan) exhibit different left ventricular ejection fraction distributions, indicating population shift. Right: models were compared on the combined external datasets (397 Kaggle and 206 Rescan scans; *n* = 603) for LVEF MAE and on the 102 complete repeated-scan pairs from the Rescan dataset for LVEF coefficient of variation (CV). Fine-tuned CineMA achieved performance comparable to nnUNet and outperformed other baselines for MAE, while outperforming all models for CV. **c** Top: models were compared on the combined test sets from ACDC, M&Ms and M&Ms2. Fine-tuned CineMA approached nnUNet and outperformed other baselines. Bottom: models were compared on the external datasets (Kaggle and Rescan). Fine-tuned CineMA achieved comparable LVEF error and lower CV. RV right ventricle, MYO myocardium, LV left ventricle, EF ejection fraction, LVEF left ventricular ejection fraction. In each box plot, the central line indicates the median, the box spans the interquartile range (25th–75th percentiles), and the whiskers extend to the 5th and 95th percentiles.

The fine-tuned CineMA was further evaluated in a zero-shot setting by segmenting the left ventricle in the Kaggle^10^ and Rescan^6^ datasets, without additional training. LVEF was then derived from maximum and minimum LV volumes over the cardiac cycle. These datasets, which were not used during pre-training or fine-tuning, represented a marked population shift, with average LVEF more than 20 percentage points higher than in the training data population (Fig. 2). Across fine-tuned variants, CineMA^FineTune^ achieved a mean absolute error of 4.65% for LVEF, comparable to nnUNet (4.30%) and outperforming other baselines (Supplementary Table 7).

The Rescan dataset consists of paired images representing two independent scans of the same subject acquired within a short interval (within one week in 96% of cases). LVEF can therefore be assumed to remain consistent across repeated acquisitions, allowing assessment of the model’s consistency using the coefficient of variation. Across fine-tuned variants, CineMA^FineTune^ achieved a coefficient of variation of 6.73% across paired scans, significantly lower than nnUNet (9.53%) and other baselines. Notably, despite not being trained on the Rescan dataset, CineMA^FineTune^ also outperformed the previously reported coefficient of variation of 8.8% by Bhuva et al.^6^. This reduction in variability suggests that pre-training acts as a robust regulariser, an advantage that becomes more pronounced in low-data settings, as examined in the next section.

### Label efficiency for segmentation and EF prediction

The prior knowledge gained during pre-training was further assessed through label efficiency, that is, the amount of labelled data required to achieve a given level of performance. By varying the proportion of training data, we found that CineMA^FineTune^ consistently outperformed both CineMA^RandInit^ and UNet^RandInit^ across all metrics and datasets, including population-shifted external cohorts (Fig. 2). Although performance gaps remain between CineMA^FineTune^ and nnUNet on the ACDC, M&Ms and M&Ms2 test splits, evaluation on the Kaggle and Rescan datasets demonstrates that CineMA^FineTune^ achieved comparable LVEF prediction accuracy to nnUNet. Using only 10% of the available training data, CineMA^FineTune^’s coefficient of variation increased modestly from 6.73 to 8.47%, whereas nnUNet’s increased from 9.53 to 20.20%. These results suggest that although nnUNet’s dataset-specific tuning strategy improves performance on similar data distributions, it may be less robust to distribution shifts. In contrast, pre-training regularises CineMA’s representation learning during fine-tuning, yielding better generalisation capacity, particularly in low-data settings. This consistency across heterogeneous acquisitions addresses a key clinical need: reducing the operator- and scanner-dependent variability that currently limits CMR interpretation, thereby supporting more standardised cardiac function assessment in multi-centre studies and longitudinal monitoring. Consequently, CineMA can be considered a viable alternative to nnUNet for clinical studies, reducing the manual annotation burden while preserving generalisation across populations.

### Cardiovascular disease classification and detection

**Fig. 3:**
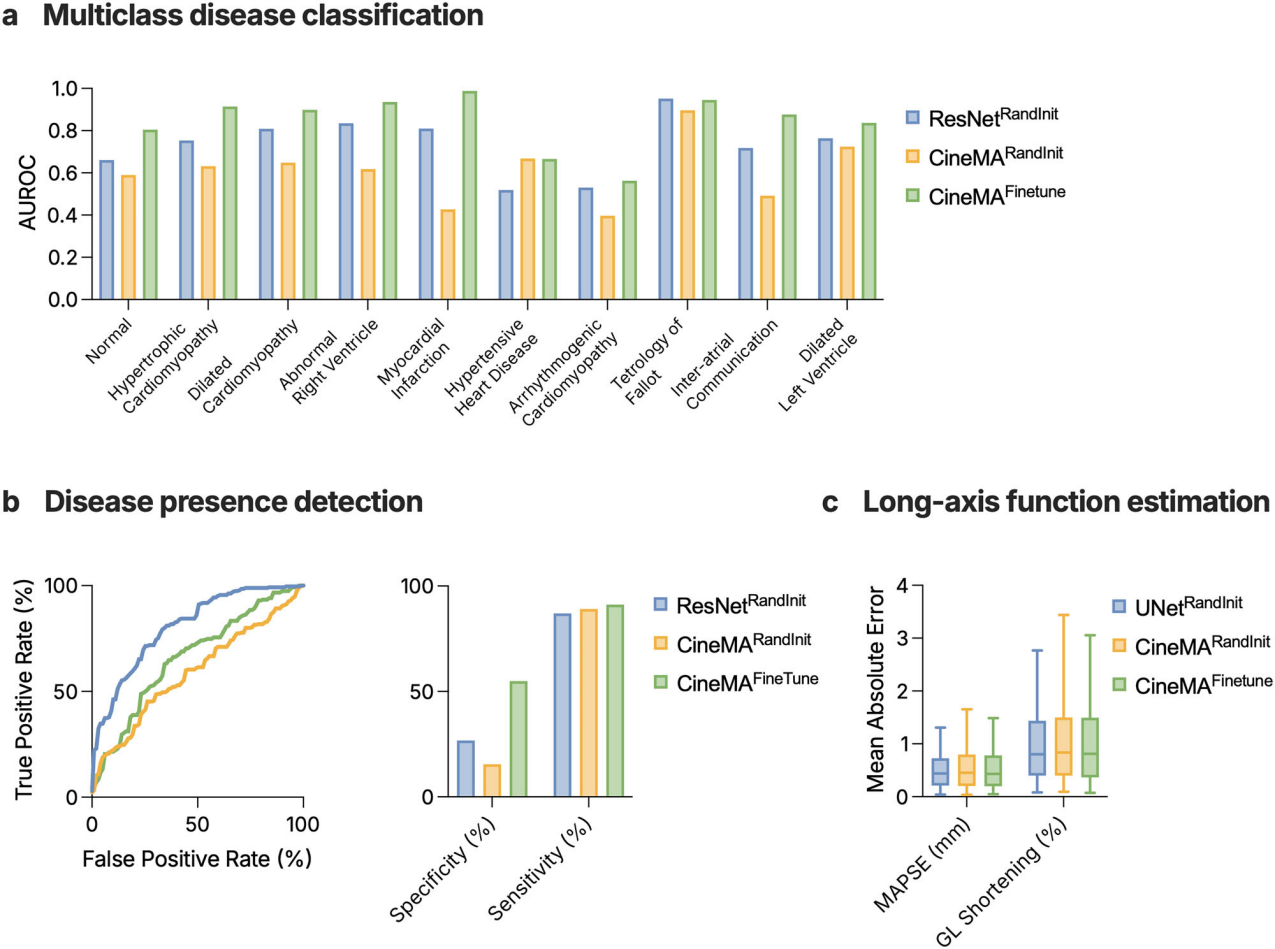
Diagnostic performance evaluation for disease classification, detection and long-axis function estimation. **a** Fine-tuned CineMA significantly outperformed ResNet^RandInit^ across nearly all cardiovascular disease and normal conditions on the combined test sets from ACDC, M&Ms and M&Ms2 (*n* = 341). **b** For binary disease detection, all disease classes were merged into a single 'disease present' category and performance was evaluated on the combined held-out SAX test splits from ACDC, M&Ms and M&Ms2 after excluding samples whose labels were absent from the corresponding training split (*n* = 263). Fine-tuned CineMA consistently achieved higher sensitivity, leading to higher AUROC values, and also demonstrated higher specificity, indicating potential to reduce over-diagnosis while maintaining a low false-negative rate. **c** Fine-tuned CineMA achieved comparable performance to UNet^RandInit^ for long-axis function estimation on the Landmark dataset (*n* = 1155, 2C and 4C images from the test set). Wilcoxon signed-rank tests between CineMA^FineTune^ and baselines showed no statistically significant differences. GLS global longitudinal shortening, MAPSE mitral annular plane systolic excursion, AUROC area under the receiver operating characteristic curve. In each box plot, the central line indicates the median, the box spans the interquartile range (25th–75th percentiles), and the whiskers extend to the 5th and 95th percentiles.

Beyond ventricle segmentation and ejection fraction estimation, we evaluated CineMA’s diagnostic performance across multiple tasks, including cardiovascular disease diagnosis and long-axis function assessment. Disease classification was performed separately on the ACDC, M&Ms and M&Ms2 datasets, each comprising distinct diagnostic labels (Table 2). CineMA^FineTune^ consistently outperformed baseline models across all three datasets (Supplementary Table 8), with gains observed across nearly all disease classes, including the commonly observed hypertrophic cardiomyopathy (Fig. 3). For binary disease detection, all cardiac disease types were merged into a single 'disease present' category, and performance was evaluated on the combined test splits. CineMA^FineTune^ achieved higher specificity (54.9% vs. 26.8%) and sensitivity (91.1% vs. 87.0%) than ResNet^RandInit^ (Supplementary Table 8). This improvement in specificity suggests CineMA^FineTune^ could reduce over-diagnosis while maintaining a low false-negative rate, supporting its potential role in automated screening and triage. CineMA^FineTune^ was also fine-tuned for image-wise classification and regression tasks, including MRI vendor, patient sex, EF, BMI and age (Supplementary Tables 9 and 10). Across all tasks, CineMA^FineTune^ demonstrated that self-supervised pre-training yields transferable representations for both clinical and acquisition-related characteristics.

### Long-axis landmark localisation and function regression

Long-axis function was assessed on two-chamber (2C) and four-chamber (4C) views using the Landmark dataset, by localising keypoints at the mitral valve and apex that define the mitral valve plane and ventricular length^11^. Heatmap-based and coordinate-based approaches were used to localise landmark locations. CineMA^FineTune^ achieved mean Euclidean distance errors of 1.00 mm across 2C and 4C views, improving on previously reported errors^11^ by at least 1 mm (Supplementary Table 11) and matching UNet^RandInit^ performance. For MAPSE estimation, CineMA^FineTune^ achieved an MAE of 0.58 mm, slightly higher than the UNet^RandInit^ baseline (0.54 mm), though the difference is clinically negligible. GLS estimation yielded similar MAEs of 1.12% for CineMA^FineTune^ and 1.08% for UNet^RandInit^. Overall, fine-tuned CineMA achieved accuracy comparable to baseline models without a statistically significant difference, indicating that it is a feasible alternative for these functional assessment tasks.

### Systemic diseases and survival prediction

**Fig. 4:**
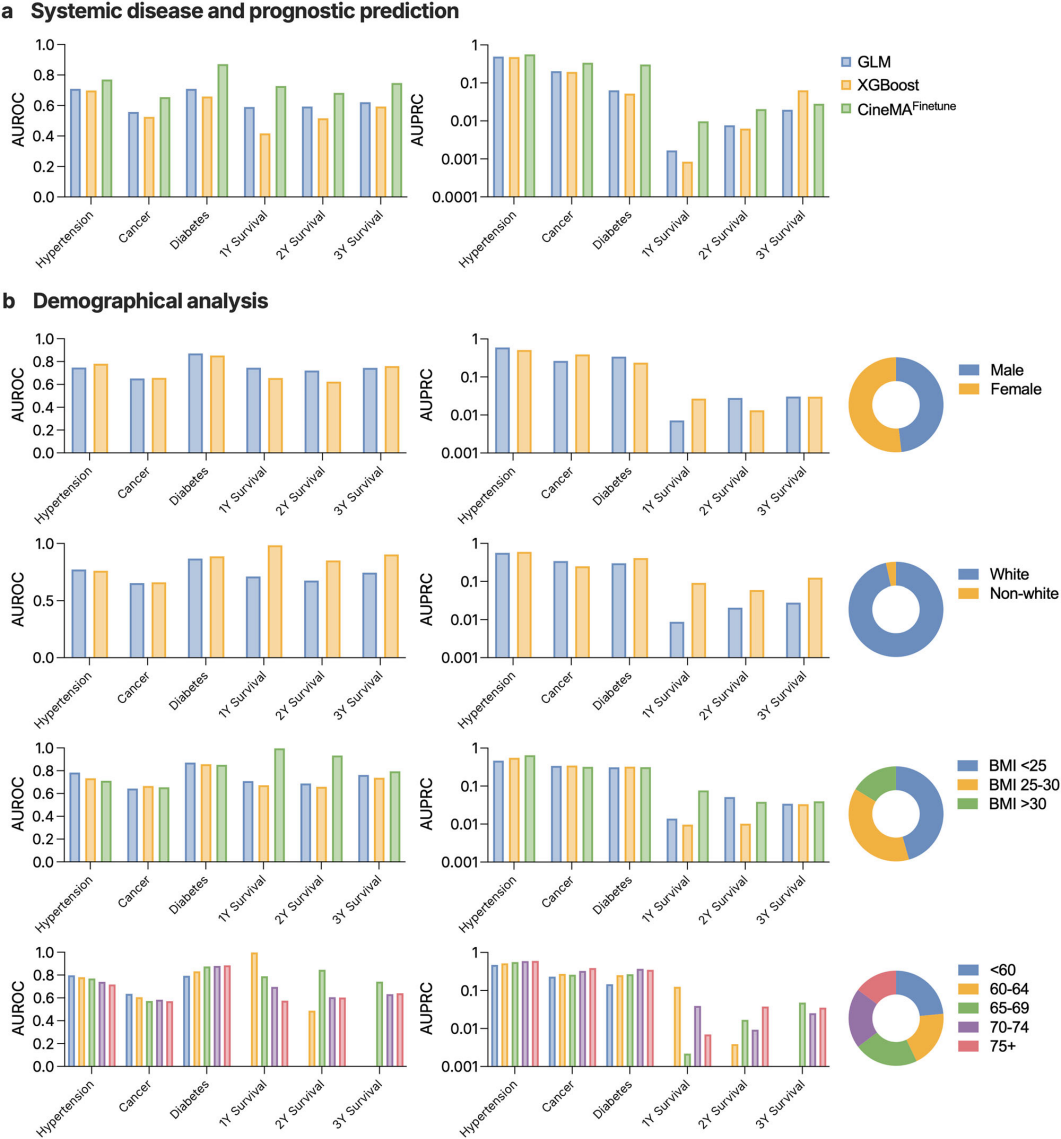
Classification performance for systemic diseases and survival prediction and demographic analyses on the UKB dataset (*n* = 30,386). **a** Fine-tuned CineMA outperformed generalised linear model (GLM) and XGBoost models over all tasks that use model-derived morphometric metrics, as measured by both AUROC (left) and AUPRC (right). **b** Model performance grouped by different demographic groups and distribution of groups. AUROC area under the receiver operating characteristic curve, AUPRC area under the precision-recall curve, BMI body mass index, UKB UK Biobank.

Following the improved classification of cardiovascular diseases, we conducted exploratory analyses to evaluate whether CineMA^FineTune^ representations could capture subtle phenotypical signatures associated with systemic comorbidities (hypertension, cancer and diabetes) or longitudinal survival (1-, 2- and 3-year horizons). To preserve learned features and reduce training time, the encoder was frozen except for the last block, enabling convergence within 20 epochs. Despite the inherent difficulty of these tasks, subtle cardiac manifestations of systemic conditions and severe class imbalance in survival prediction, CineMA^FineTune^ achieved AUROCs of 0.771 for hypertension, 0.655 for cancer, and 0.871 for diabetes, and AUROCs of 0.727, 0.682 and 0.748 for 1-, 2- and 3-year survival, respectively (Supplementary Table 12). CineMA^FineTune^ also achieved higher AUPRC than baselines for all systemic disease tasks (0.566 for hypertension, 0.341 for cancer and 0.305 for diabetes). For survival prediction, AUPRC remained low across all models due to extreme class imbalance (positive rates of 0.10–1.19%). CineMA^FineTune^ outperformed baselines across all tasks except 3-year survival AUPRC, where XGBoost achieved a higher score (0.064 vs. 0.028) despite a substantially lower AUROC (0.593 vs. 0.748). However, as shown in the precision-recall curves (Supplementary Fig. 3), XGBoost’s precision falls below that of CineMA^FineTune^ beyond 10% recall. Across all tasks, CineMA^FineTune^ consistently outperformed GLM and XGBoost baselines trained on model-derived morphometric metrics, including ventricular volumes and ejection fraction from SAX views alongside ventricular length and function estimations from LAX views (Fig. 4 and Supplementary Fig. 3). This suggests that the latent representations encode prognostically relevant information beyond conventional morphometric parameters, demonstrating the potential of foundation models to support clinical risk stratification. Validation in external cohorts will be an important next step toward clinical translation.

We further assessed whether the model’s accuracy varies across demographic subgroups (sex, race, BMI and age). As shown in Fig. 4, the fine-tuned CineMA model demonstrates comparable performance across sex, race and BMI groups when predicting systemic diseases. Notably, despite UKB data predominantly consisting of White subjects (90.86%), we did not observe significant performance deterioration in non-White groups, despite the class imbalances (Table 3). For groups with different ages, we observed changes in performance between groups, indicating potential room for investigation in future studies.

## Discussion

In this study, we introduced CineMA, a cine CMR foundation model developed for a broad range of clinically relevant tasks. CineMA is a multi-view conv-transformer masked autoencoder that was pre-trained on over 15 million CMR images and evaluated on eight independent datasets. Without extensive hyperparameter tuning per dataset, CineMA demonstrated competitive segmentation accuracy to nnUNet and more consistent estimates of ejection fraction, particularly in low-data scenarios and in external datasets with population shifts. Task-specific optimisation of hyperparameters, including the data augmentation strategy, training schedule, and prediction head architecture, may further close this gap and is left for future work. CineMA also performed well in cardiovascular disease detection and diagnosis, achieving substantially higher specificity and increased sensitivity compared to the baseline methods, potentially reducing over-diagnosis. We further evaluated fine-tuned CineMA for systemic disease and survival prediction, achieving non-trivial performance despite severe class imbalance. These findings require validation in external cohorts before clinical application. Through this comprehensive benchmark, we demonstrated the value of the proposed cine CMR foundation models across a diverse set of applications in reducing training data requirements and improving performance, consistency and generalisability. These findings highlight the advantages of fine-tuning foundation models as an alternative to task-specific training and their potential to enable cardiac imaging applications in settings previously constrained by limited data or suboptimal model performance.

Multiple prior efforts have explored cardiac foundation models by fine-tuning existing models^17^ or pre-training new ones^18,19^. These models have evolved from 2D to multi-view architectures, with dataset sizes growing to as many as 42,000 CMR studies. CineMA advances this direction by incorporating convolutional layers to reduce memory consumption and improve performance, and by scaling pre-training to over 74,000 CMR studies comprising more than 15 million images. While these works have demonstrated preliminary evidence of the benefits of foundation models, their evaluations were often fragmented, performed under varying conditions, and lacked standardisation in baselines, metrics, or datasets. In particular, some omitted clinically relevant metrics, lacked strong convolutional neural network baselines, or were not validated on external datasets. To address these gaps, our study consolidated a broad set of clinically relevant tasks and extended the benchmark to include more external datasets, assess additional metrics, such as prediction consistency across repeated scans, and systematically examine label efficiency by varying training data sizes. Combined with systematic comparisons against strong convolutional baselines, including nnUNet and ResNet, CineMA’s consistent performance demonstrates its robustness and broad applicability.

Nevertheless, there remain several limitations and challenges requiring further exploration. First, although CineMA was evaluated on non-cardiac conditions and survival prediction, these analyses were limited to UKB data and did not include cardiac clinical outcomes such as major adverse cardiovascular events. Future work may also evaluate outcome-prediction models on additional clinical endpoints and external cohorts to determine the added value of CineMA for improving prognostic models and risk-stratification pipelines. The systemic disease predictions in this study reflect prevalent conditions; investigating incident disease prediction, where the model identifies individuals who will subsequently develop a condition, would be a clinically valuable extension. The diabetes and hypertension labels were self-reported via touchscreen questionnaire and subject to recall bias. Future work may incorporate International Classification of Diseases (ICD) codes to obtain more reliable labels. Second, CineMA was pre-trained exclusively on cine CMR images from the UKB^30^. Expanding the pre-training dataset to include additional sources could increase population and acquisition diversity, potentially improving generalisability. In addition, we observed limited benefit of CineMA on LGE and T2-weighted images (Supplementary Tables 13 and 14), consistent with the absence of these modalities during pre-training. T2-weighted imaging, including T2-STIR sequences critical for detecting myocardial oedema in myocarditis and acute cardiac injury, was only available in the limited MyoPS2020 dataset (*n* = 45). Furthermore, the evaluation datasets did not include myocarditis or other inflammatory cardiomyopathies, nor valvular pathologies, such as aortic or mitral regurgitation, which can be assessed using cine CMR in clinical practice. It would also be valuable to develop modality-specific foundation models^31^ or perform continued unsupervised pre-training on LGE, T2-weighted, and other non-cine CMR sequences, and implement modality-specific adapters to bridge the distributional gap. Third, while the proposed conv-transformer architecture yielded strong performance when fine-tuned, it did not consistently match purely convolutional architectures when trained from random initialisation. This performance gap was also observed in previous work^18,19,32^. Future work is required to optimise the architectural design. Finally, training foundation models requires substantial computational resources, particularly due to the large parameter counts in transformer-based architectures. This presents a practical barrier to adoption in clinical environments. Exploring more efficient architectures, such as mixture-of-experts models^33^, could help mitigate this constraint and enhance scalability.

To facilitate clinical translation and lower the computational barriers to foundation model development, we have released the source code and weights for all pre-trained and fine-tuned CineMA models. For practical implementation, we propose a stratified deployment strategy. Practitioners should prioritise direct inference for short-axis segmentation using the M&Ms2 fine-tuned model, which was trained on the largest dataset and demonstrates the best performance on unseen data distributions. Prediction stability can be further enhanced through model ensembling across different training seeds and datasets. In particular, these segmentation models can be used to estimate ventricle volumes and thereby label end-diastolic and end-systolic frames, which could help reduce the required amount of labelled training data. In scenarios where local labels are available, CineMA serves as a robust alternative to nnUNet, particularly in low-data regimes where pre-training provides a regularisation effect. For disease detection and classification, the released weights enable immediate inference when the target pathologies align with existing cohorts. Fine-tuning the CineMA backbone is recommended over standard ResNet architectures for novel conditions. For long-axis functional estimation, the provided weights allow initial zero-shot validation on custom cohorts prior to adaptation. For downstream tasks not evaluated in this study, such as image registration or super-resolution, the CineMA encoders can function as pre-trained feature extractors, as their latent embeddings capture complex cardiac geometry that can be effectively transferred to predict velocity fields or recover sub-pixel anatomical details^34^. Finally, for population-level studies, CineMA models enable high-throughput derivation of digital biomarkers, including cardiac functional indices and estimates of cardiac and systemic disease. When integrated into prognostic models or risk stratification pipelines, these CineMA-derived metrics facilitate complex multi-modal association studies while providing image-based interpretability for downstream clinical predictions.

## Conclusion

We demonstrated the efficacy and efficiency of CineMA compared to traditional task-specific convolutional models across a range of clinically relevant tasks, offering a strong alternative to existing methods. The public release of the full training and evaluation pipeline, along with model weights, democratises access to rigorous evaluation, modular frameworks, and high-performance models that would otherwise require substantial development effort and computational resources. Together with the suggested deployment strategy, we expect to lower the barrier to clinical and commercial adoption, enabling institutions worldwide to benefit from our work and accelerate research in cardiovascular diagnostics.

## Supplementary information

Transparent Peer Review File

Supplementary Material

Description of Additional Supplementary Files

Supplementary Data 1

## Data Availability

Source data for Figs. 2–4 can be accessed from Supplementary Data 1. The UK Biobank cardiac MRI data can be accessed by bona fide researchers through an application to the UK Biobank (https://www.ukbiobank.ac.uk). The ACDC dataset is available at https://www.creatis.insa-lyon.fr/Challenge/acdc/databases.html. The M&Ms and M&Ms-2 datasets can be requested at https://www.ub.edu/mnmsand https://www.ub.edu/mnms-2/. The Kaggle cardiac MRI dataset is available at https://www.kaggle.com/c/second-annual-data-science-bowl/data. The EMIDEC dataset is available at https://emidec.com. The MyoPS2020 dataset is available at https://zmiclab.github.io/zxh/0/myops20/. The Landmark dataset^11^ is not publicly available, but investigators from research institutions may request access by contacting the authors directly. Rescan data is available at https://thevolumesresource.com/.

## Code availability

All code for pre-training, fine-tuning and evaluation, along with weights of pre-trained and fine-tuned models, are publicly available under an MIT license at https://github.com/mathpluscode/CineMA.
