# Supplementary material for: Development and validation of a versatile foundation model for cine cardiac magnetic resonance image analysis
Source: Commun Med (Lond). 2026 May 13;6:408. doi: 10.1038/s43856-026-01636-0 (PMC13396196; doi:10.1038/s43856-026-01636-0)
Supplement: Supplementary file 2 — Supplementary Material [file 43856_2026_1636_MOESM2_ESM.pdf]

Supplementary Material: Development and validation of a versatile foundation model for cine cardiac magnetic resonance image analysis

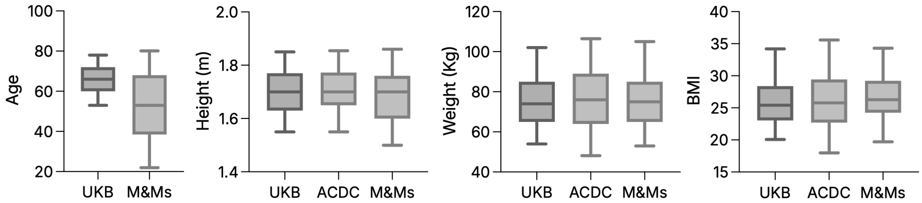

Fig. 1 Demographic distribution between UK Biobank (UKB) data, ACDC and M&Ms datasets on available labels. BMI, body mass index. In each box plot, the central line indicates the median, the box spans the interquartile range (25th–75th percentiles), and the whiskers extend to the 5th and 95th percentiles.

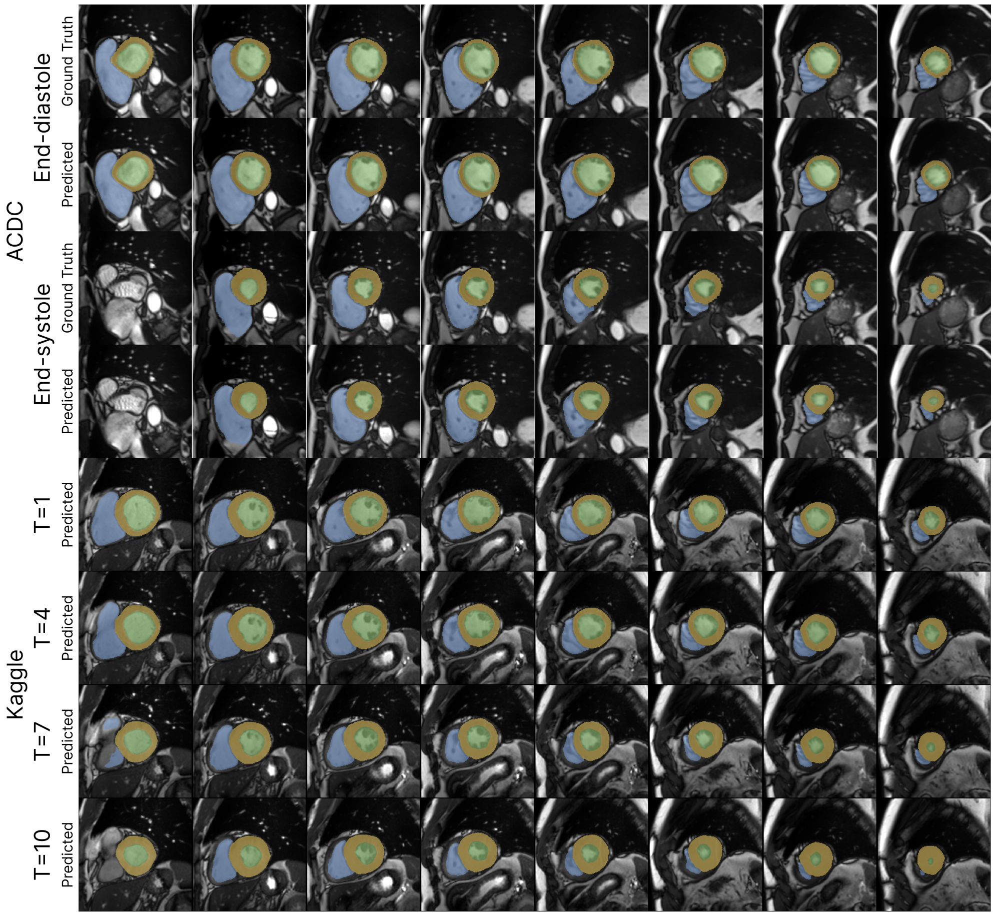

Fig. 2 Example segmentations on ACDC and Kaggle dataset test splits. The segmentation model was a fine-tuned version of CineMA using ACDC dataset. The model was not trained on Kaggle dataset. Each column corresponds to a short-axis slice.

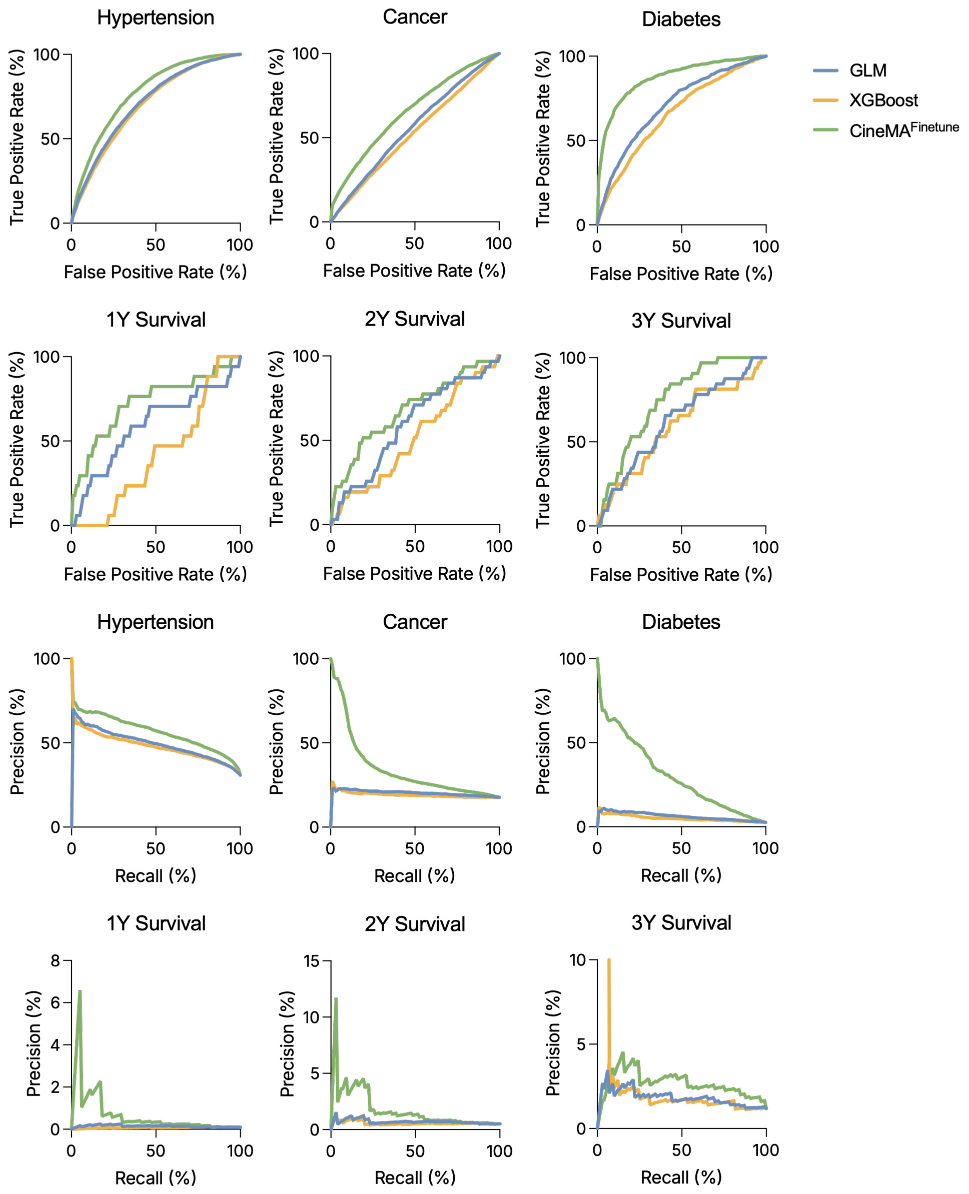

Fig. 3 Receiver operating characteristic curves and precision-recall curves for fine-tuned CineMA models and baselines on systemic disease and survival predictions. GLM, Generalised Linear Model.

Table 1 Hyperparameters for downstream task model training. If the training dataset size is smaller than the preset batch size, the batch size is reduced to the closest power of two. CE, cross-entropy. MSE, mean squared error. MCC, Matthews correlation coefficient. AUROC, area under the receiver operating characteristic curve.

Hyperparameter	Classification	Regression	Segmentation
Total Epochs	800	800	4000
Warmup Epochs	10	10	50
Peak Learning Rate	0.001	0.001	0.001
End Learning Rate	10^{-5}	10^{-5}	10^{-5}
Batch Size	64	64	64
Loss	CE Loss	MSE	Dice Loss + CE Loss
Augmentation	Contrast adjustment, Gaussian noise, Affine transformation.	Contrast adjustment, Gaussian noise, Affine transformation.	Contrast adjustment, Gaussian noise, Affine transformation, Crop dropout.
Validation Frequency	20	20	100
Validation Patience	5	5	5
Validation Metric	MCC	Absolute Error	Dice Score
Hyperparameter	Landmark localisation<br>Heatmap	 Coordinate	Systemic disease<br>Survival
Total Epochs	400	400	20
Warmup Epochs	10	10	2
Peak Learning Rate	0.001	0.001	0.001
End Learning Rate	10^{-5}	10^{-5}	10^{-5}
Batch Size	64	64	256
Loss	Dice Loss + CE Loss	Wing Loss [1]	Focal Loss
Augmentation	Contrast adjustment, Gaussian noise, Affine transformation.	Contrast adjustment, Gaussian noise, Affine transformation.	Contrast adjustment, Gaussian noise, Affine transformation.
Validation Frequency	20	20	1
Validation Patience	5	5	5
Validation Metric	L^2 distance	L^2 distance	AUROC

Table 2 Ablation of segmentation model architecture on ACDC, M&Ms, and M&Ms2 datasets. All models were trained from randomly initialised weights. RV, right ventricle. MYO, myocardium. LV, left ventricle. Values are shown as mean ± std across samples.

Data	Model	Input patch size	Dice Score (%) RV	MYO	LV	Mean
ACDC	w/ Conv	4	**90.12**± 6.01	**86.52**± 7.18	**92.08**± 8.55	**89.57**± 5.79
	w/o Conv	16	85.66± 9.06	81.45± 5.65	89.55± 8.09	85.55± 5.49
M&Ms	w/ Conv	4	**84.70**± 9.56	**81.16**± 6.50	**89.24**± 6.99	**85.03**± 6.05
	w/o Conv	16	80.58± 14.38	75.47± 8.19	85.76± 10.05	80.60± 8.88
M&Ms2	w/ Conv	4	**84.97**± 13.54	**81.92**± 8.64	**91.00**± 9.15	**85.96**± 9.12
	w/o Conv	16	81.44± 15.55	76.79± 9.73	88.58± 10.30	82.27± 10.29

Data	Model	Input patch size	95% Hausdorff Distance (mm) RV	MYO	LV	Mean
ACDC	w/ Conv	4	**5.43**± 4.52	**4.66**± 6.34	**4.97**± 9.00	**4.99**± 5.50
	w/o Conv	16	7.87± 6.05	5.42± 4.23	5.81± 4.88	6.37± 4.15
M&Ms	w/ Conv	4	**10.25**± 11.40	**6.24**± 3.82	**6.80**± 4.16	**7.76**± 5.04
	w/o Conv	16	10.33± 8.85	8.44± 6.82	9.36± 8.59	9.38± 6.70
M&Ms2	w/ Conv	4	14.14± 16.65	**7.47**± 5.50	**7.46**± 5.68	**9.69**± 7.36
	w/o Conv	16	**12.89**± 12.94	8.78± 8.21	8.74± 8.80	10.04± 7.75

Table 3 Ventricle and myocardium segmentation performance in Dice score. SAX dataset represents the combined test sets from ACDC, M&Ms, and M&Ms2 on SAX views. For baselines on ACDC, M&Ms, and M&Ms2, the range is calculated from the top five methods. SAX, short-axis. LAX, long-axis. 4C, four-chamber. RV, right ventricle. MYO, myocardium. LV, left ventricle. Values are shown as mean ± std across samples.

Data	Model	Dice Score (%)			
		RV	MYO	LV	Mean
ACDC (SAX)	UNet^{RandInit}	89.30± 6.78	86.69± 5.29	92.62± 5.39	89.54± 4.25
	CineMA^{RandInit}	90.12± 6.01	86.52± 7.18	92.08± 8.55	89.57± 5.79
	CineMA^{FineTune}	91.07± 4.79	87.94± 4.68	**93.63**± 4.18	90.88± 2.90
	nnUNet	**91.57**± 5.17	**88.94**± 4.57	93.25± 5.84	**91.26**± 3.36
	Jacob et al. [2]	90.7± 18	87.9± 14	93.3± 14	90.63± 15
	ACDC [3]	[90.70, 92.25]	[88.50, 91.05]	[93.10, 94.95]	[91.07, 92.75]
M&Ms (SAX)	UNet^{RandInit}	86.21± 9.47	82.13± 6.40	89.67± 7.25	86.01± 5.79
	CineMA^{RandInit}	84.70± 9.56	81.16± 6.50	89.24± 6.99	85.03± 6.05
	CineMA^{FineTune}	87.86± 7.32	83.39± 5.38	**90.71**± 5.38	87.32± 4.08
	nnUNet	**88.04**± 7.29	**83.60**± 5.96	90.54± 6.55	**87.39**± 4.76
	M&Ms[4]	[85.75, 88.50]	[83.35, 85.30]	[90.30, 91.25]	[86.62, 88.35]
M&Ms2 (SAX)	UNet^{RandInit}	88.26± 7.44	83.55± 6.07	92.20± 5.43	88.00± 5.23
	CineMA^{RandInit}	84.97± 13.54	81.92± 8.64	91.00± 9.15	85.96± 9.12
	CineMA^{FineTune}	89.76± 6.14	84.82± 4.92	93.11± 3.76	89.23± 3.57
	nnUNet	**91.20**± 5.63	**85.61**± 5.51	**93.70**± 3.81	**90.17**± 3.76
	M&Ms2 [5]	[92.00, 92.70]			
SAX	UNet^{RandInit}	87.61± 8.29	83.45± 6.27	91.27± 6.33	87.44± 5.47
	CineMA^{RandInit}	85.62± 11.35	82.30± 7.84	90.47± 8.33	86.13± 7.72
	CineMA^{FineTune}	89.21± 6.56	84.72± 5.28	92.24± 4.68	88.72± 3.90
	nnUNet	**90.01**± 6.47	**85.31**± 5.84	**92.40**± 5.54	**89.24**± 4.40
M&Ms2 (LAX 4C)	UNet^{RandInit}	90.51± 8.12	86.73± 7.84	94.67± 8.38	90.63± 7.49
	CineMA^{RandInit}	89.88± 9.45	86.84± 6.93	94.77± 8.49	90.50± 7.52
	CineMA^{FineTune}	91.08± 5.56	87.68± 5.99	95.62± 4.56	91.46± 4.26
	nnUNet	**91.51**± 6.99	**88.04**± 7.65	**95.65**± 6.26	**91.73**± 5.96

Table 4 Ventricle and myocardium segmentation performance in Hausdorff distance. SAX dataset represents the combined test sets from ACDC, M&Ms, and M&Ms2 on SAX views. For baselines on ACDC and M&Ms, the range is calculated from the top five methods. SAX, short-axis. LAX, long-axis. 4C, four-chamber. RV, right ventricle. MYO, myocardium. LV, left ventricle. Values are shown as mean ± std across samples.

Data	Model	95% Hausdorff Distance (mm)			
		RV	MYO	LV	Mean
ACDC (SAX)	UNet^{RandInit}	5.41± 4.63	3.98± 4.03	3.90± 4.08	4.43± 3.44
	CineMA^{RandInit}	5.43± 4.52	4.66± 6.34	4.97± 9.00	4.99± 5.50
	CineMA^{FineTune}	**4.27**± 3.56	**2.98**± 2.87	**3.01**± 3.51	**3.42**± 2.49
	nnUNet	4.53± 4.36	3.64± 3.40	3.48± 3.69	3.88± 2.78
M&Ms (SAX)	UNet^{RandInit}	6.77± 4.88	6.07± 6.11	6.29± 6.14	6.36± 4.49
	CineMA^{RandInit}	10.25± 11.40	6.24± 3.82	6.80± 4.16	7.76± 5.04
	CineMA^{FineTune}	5.79± 4.26	**5.14**± 3.74	**4.99**± 3.67	**5.31**± 2.85
	nnUNet	**5.66**± 4.41	5.39± 6.17	5.32± 5.66	5.46± 4.12
M&Ms2 (SAX)	UNet^{RandInit}	8.56± 6.67	6.51± 4.88	6.39± 4.68	7.15± 4.28
	CineMA^{RandInit}	14.14± 16.65	7.47± 5.50	7.46± 5.68	9.69± 7.36
	CineMA^{FineTune}	7.11± 6.67	5.30± 3.89	5.27± 3.88	5.89± 3.50
	nnUNet	**5.30**± 4.27	**4.91**± 4.14	**4.86**± 4.22	**5.02**± 3.14
SAX	UNet^{RandInit}	7.40± 5.85	5.97± 5.36	5.98± 5.29	6.44± 4.34
	CineMA^{RandInit}	11.33± 13.81	6.58± 5.14	6.84± 5.84	8.24± 6.47
	CineMA^{FineTune}	6.18± 5.52	**4.89**± 3.78	**4.83**± 3.82	5.30± 3.23
	nnUNet	**5.33**± 4.35	4.91± 4.98	4.84± 4.81	**5.03**± 3.54
M&Ms2 (LAX 4C)	UNet^{RandInit}	5.93± 6.47	4.34± 6.89	4.25± 6.00	4.84± 5.73
	CineMA^{RandInit}	6.68± 8.69	4.46± 6.63	4.20± 5.87	5.11± 6.29
	CineMA^{FineTune}	5.21± 3.44	3.58± 4.64	3.38± 3.66	4.06± 3.30
	nnUNet	**2.43**± 3.88	**3.16**± 6.60	**1.16**± 4.42	**2.25**± 4.36

Table 5 Ventricle and myocardium segmentation performance in volume prediction. SAX dataset represents the combined test sets from ACDC, M&Ms, and M&Ms2 on SAX views. SAX, short-axis. RV, right ventricle. MYO, myocardium. LV, left ventricle. MAE, mean absolute error. Values are shown as mean ± std across samples.

Data	Model	Volume MAE (ml) RV	MYO	LV	Mean
ACDC (SAX)	UNet^{RandInit}	11.25± 11.27	8.14± 7.08	5.78± 4.67	8.39± 5.41
	CineMA^{RandInit}	8.81± 9.92	8.24± 11.42	6.16± 4.86	7.74± 6.04
	CineMA^{FineTune}	9.19± 10.34	7.59± 5.75	**5.06**± 3.59	7.28± 4.27
	nnUNet	**8.35**± 10.54	**7.11**± 5.83	5.46± 4.27	**6.98**± 4.46
M&Ms (SAX)	UNet^{RandInit}	10.46± 9.10	13.61± 11.82	9.42± 8.90	11.16± 6.73
	CineMA^{RandInit}	12.64± 10.89	12.83± 9.45	8.77± 7.27	11.41± 5.95
	CineMA^{FineTune}	**9.24**± 9.16	**11.47**± 8.86	**8.50**± 7.44	**9.74**± 5.61
	nnUNet	10.10± 10.03	12.81± 12.38	8.98± 8.52	10.63± 7.24
M&Ms2 (SAX)	UNet^{RandInit}	12.61± 11.55	8.14± 6.57	8.17± 7.36	9.64± 5.68
	CineMA^{RandInit}	17.38± 22.10	8.61± 7.27	9.03± 7.33	11.67± 9.14
	CineMA^{FineTune}	12.45± 13.24	**7.31**± 6.13	7.46± 6.34	9.08± 5.71
	nnUNet	**9.87**± 10.00	7.53± 6.29	**7.25**± 7.08	**8.21**± 5.09
	M&Ms2 [5]	[8.89, 9.79]			
SAX	UNet^{RandInit}	11.57± 10.64	10.29± 9.44	8.31± 7.78	10.06± 6.15
	CineMA^{RandInit}	14.26± 17.18	10.22± 9.10	8.51± 7.06	10.99± 7.71
	CineMA^{FineTune}	10.71± 11.48	**8.99**± 7.54	**7.52**± 6.58	9.07± 5.53
	nnUNet	**9.74**± 10.09	9.54± 9.49	7.67± 7.46	**8.98**± 6.11

Table 6 Ejection fraction (EF) regression performance using segmentation models. The SAX dataset represents the combined test set from ACDC, M&Ms, and M&Ms2 on SAX views. For baselines on M&Ms2, the range is calculated from the top five methods. SAX, short-axis. LAX, long-axis. 4C, four-chamber. RV, right ventricle. LV, left ventricle. EF, ejection fraction. LVEF, left ventricular ejection fraction. RVEF, right ventricular ejection fraction. MAE, mean absolute error. Values are shown as mean ± std across samples.

Dataset	Model	LVEF MAE (%)	RVEF MAE (%)
ACDC (SAX)	$\text{UNet}^{\text{RandInit}}$	**2.20**± 1.79	5.15± 4.49
	$\text{CineMA}^{\text{RandInit}}$	2.80± 2.87	4.37± 4.60
	$\text{CineMA}^{\text{FineTune}}$	2.23± 2.19	3.82± 3.45
	nnUNet	2.96± 2.79	**3.74**± 3.87
	ACDC [3]	[2.1, 2.6]	[4.7, 6.3]
M&Ms (SAX)	$\text{UNet}^{\text{RandInit}}$	4.71± 5.30	5.94± 5.10
	$\text{CineMA}^{\text{RandInit}}$	4.39± 4.24	6.47± 5.64
	$\text{CineMA}^{\text{FineTune}}$	**4.08**± 3.52	**5.87**± 5.45
	nnUNet	4.63± 4.58	6.12± 5.78
M&Ms2 (SAX)	$\text{UNet}^{\text{RandInit}}$	3.22± 2.96	6.34± 6.93
	$\text{CineMA}^{\text{RandInit}}$	4.08± 4.83	8.13± 8.58
	$\text{CineMA}^{\text{FineTune}}$	3.52± 2.98	5.85± 5.41
	nnUNet	**3.02**± 2.82	**4.82**± 4.42
	M&Ms2 [5]		[4.36, 4.67]
SAX	$\text{UNet}^{\text{RandInit}}$	3.65± 4.04	6.01± 5.94
	$\text{CineMA}^{\text{RandInit}}$	4.02± 4.38	6.93± 7.14
	$\text{CineMA}^{\text{FineTune}}$	**3.55**± 3.16	5.56± 5.23
	nnUNet	3.64± 3.70	**5.17**± 5.00
M&Ms2 (LAX 4C)	$\text{UNet}^{\text{RandInit}}$	2.91± 3.02	5.88± 5.87
	$\text{CineMA}^{\text{RandInit}}$	3.01± 4.61	6.25± 7.18
	$\text{CineMA}^{\text{FineTune}}$	**2.78**± 2.78	**5.31**± 4.70
	nnUNet	3.17± 3.43	5.72± 5.71

Table 7 Zero-shot performance of segmentation models on left ventricular ejection fraction regression using short-axis images. Models were trained on ACDC, M&Ms, and M&Ms2, respectively, then evaluated on Kaggle and Rescan. When the training set includes all three datasets, statistics are calculated across nine models with different training datasets and seeds. The last four rows correspond to a combined test set from Kaggle and Rescan. Vol MAE, average mean absolute error for end-diastolic and end-systolic volumes. EF, ejection fraction. MAE, mean absolute error. CV, coefficient of variation. Values are shown as mean ± std across samples.

Test	Train	Model	Vol MAE (ml)	EF MAE (%)	EF CV (%)
Kaggle	ACDC	UNet^{RandInit}	16.88± 19.17	6.24± 5.69	
		CineMA^{RandInit}	13.65± 15.89	5.98± 6.61	
		CineMA^{FineTune}	13.68± 16.37	**5.21**± 4.00	
		nnUNet	**13.08**± 18.52	5.35± 5.82	
	M&Ms	UNet^{RandInit}	16.29± 17.47	5.05± 6.13	
		CineMA^{RandInit}	15.99± 18.09	5.90± 7.07	
		CineMA^{FineTune}	15.03± 17.38	**3.83**± 3.59	
		nnUNet	**13.96**± 15.93	4.00± 4.18	
	M&Ms2	UNet^{RandInit}	11.36± 14.61	4.15± 5.33	
		CineMA^{RandInit}	12.53± 16.58	5.32± 7.35	
		CineMA^{FineTune}	11.67± 15.47	3.77± 4.03	
		nnUNet	**11.29**± 15.57	**3.70**± 4.52	
	ACDC M&Ms M&Ms2	UNet^{RandInit}	14.85± 17.39	5.15± 5.79	
		CineMA^{RandInit}	14.06± 16.93	5.74± 7.02	
		CineMA^{FineTune}	13.46± 16.51	**4.27**± 3.93	
		nnUNet	**12.78**± 16.75	4.35± 4.94	
	UKB	Shad et al. [6]		6.88	
Rescan	ACDC	UNet^{RandInit}	17.53± 21.30	7.69± 8.01	17.42
		CineMA^{RandInit}	24.29± 23.97	7.18± 5.78	10.26
		CineMA^{FineTune}	34.58± 23.39	8.43± 6.25	**9.79**
		nnUNet	**17.17**± 14.95	**3.89**± 5.61	12.67
	M&Ms	UNet^{RandInit}	**9.50**± 8.40	**3.62**± 3.87	6.87
		CineMA^{RandInit}	11.09± 10.37	4.04± 3.92	**6.71**
		CineMA^{FineTune}	10.09± 7.98	3.98± 3.91	7.39
		nnUNet	11.35± 5.75	4.33± 3.96	8.02
	M&Ms2	UNet^{RandInit}	18.12± 10.31	4.43± 4.23	8.50
		CineMA^{RandInit}	21.89± 21.13	4.66± 5.47	10.44
		CineMA^{FineTune}	**17.58**± 9.54	**3.95**± 3.65	**6.51**
		nnUNet	20.87± 8.02	4.41± 3.39	6.92
	ACDC M&Ms M&Ms2	UNet^{RandInit}	**13.04**± 11.13	4.80± 5.12	10.24
		CineMA^{RandInit}	18.35± 16.24	5.13± 4.81	8.14
		CineMA^{FineTune}	20.19± 11.09	5.22± 4.03	**6.73**
		nnUNet	16.46± 11.05	**4.21**± 4.42	9.53
	Rescan	Bhuva et al. [7]	9± 9	4± 4	8.8
Kaggle Rescan	ACDC M&Ms M&Ms2	UNet	15.23± 16.26	5.46± 5.68	
		CineMA^{RandInit}	15.96± 18.23	5.65± 6.37	
		CineMA^{FineTune}	16.07± 16.97	4.65± 4.07	
		nnUNet	**14.03**± 15.16	**4.30**± 4.77	

Table 8 Cardiovascular disease classification performance on different training datasets. SAX, short-axis. LAX, long-axis. 4C, four-chamber. AUROC, area under the receiver operating characteristic curve.

Data	Model	Classification		Detection	
		AUROC	F1 (%)	Specificity (%)	Sensitivity (%)
ACDC (SAX)	$\text{ResNet}^{\text{RandInit}}$	0.847	58.20	50.00	82.50
	$\text{CineMA}^{\text{RandInit}}$	0.629	11.90	0.00	**100.00**
	$\text{CineMA}^{\text{FineTune}}$	**0.992**	**87.75**	**90.00**	97.50
M&Ms (SAX)	$\text{ResNet}^{\text{RandInit}}$	0.682	25.67	25.81	83.78
	$\text{CineMA}^{\text{RandInit}}$	0.575	16.90	0.00	**100.00**
	$\text{CineMA}^{\text{FineTune}}$	**0.810**	**39.84**	**64.52**	89.19
M&Ms2 (SAX)	$\text{ResNet}^{\text{RandInit}}$	0.716	33.37	20.00	**92.31**
	$\text{CineMA}^{\text{RandInit}}$	0.622	29.48	**36.67**	73.08
	$\text{CineMA}^{\text{FineTune}}$	**0.801**	**50.12**	33.33	89.74
SAX	$\text{ResNet}^{\text{RandInit}}$			26.8	87.0
	$\text{CineMA}^{\text{RandInit}}$			15.5	89.1
	$\text{CineMA}^{\text{FineTune}}$			**54.9**	**91.1**
M&Ms2 (LAX 4C)	$\text{ResNet}^{\text{RandInit}}$	0.752	39.51	**46.67**	74.36
	$\text{CineMA}^{\text{RandInit}}$	0.665	23.96	36.67	70.51
	$\text{CineMA}^{\text{FineTune}}$	**0.843**	**50.45**	26.67	**91.03**

Table 9 Direct image classification performance on vendor and sex. SAX, short-axis. LAX, long-axis. 4C, four-chamber. AUROC, area under the receiver operating characteristic curve.

Target	Data	Model	AUROC	F1 (%)
Vendor	M&Ms2 (SAX)	$\text{ResNet}^{\text{RandInit}}$	**0.823**	55.96
		$\text{CineMA}^{\text{RandInit}}$	0.822	62.59
		$\text{CineMA}^{\text{FineTune}}$	0.783	**62.61**
	M&Ms2 (LAX 4C)	$\text{ResNet}^{\text{RandInit}}$	**0.841**	**64.94**
		$\text{CineMA}^{\text{RandInit}}$	0.814	59.90
		$\text{CineMA}^{\text{FineTune}}$	0.778	64.26
Sex	M&Ms (SAX)	$\text{ResNet}^{\text{RandInit}}$	0.895	75.00
		$\text{CineMA}^{\text{RandInit}}$	0.761	20.69
		$\text{CineMA}^{\text{FineTune}}$	**0.993**	**96.97**

Table 10 Direct image regression performance for left ventricular ejection fraction (LVEF), body mass index (BMI), and age. SAX, short-axis. LAX, long-axis. 4C, four-chamber. MAE, mean absolute error. Values are shown as mean ± std across samples.

Target	Data	Model	MAE
LVEF	ACDC (SAX)	ResNet^{RandInit}	5.32± 4.49
		CineMA^{RandInit}	11.08± 5.65
		CineMA^{FineTune}	**4.83**± 5.03
	M&Ms (SAX)	ResNet^{RandInit}	6.53± 5.82
		CineMA^{RandInit}	7.16± 6.68
		CineMA^{FineTune}	**5.22**± 4.41
	M&Ms2 (SAX)	ResNet^{RandInit}	7.65± 6.28
		CineMA^{RandInit}	6.65± 5.74
		CineMA^{FineTune}	**4.98**± 4.41
	M&Ms2 (LAX 4C)	ResNet^{RandInit}	6.91± 6.60
		CineMA^{RandInit}	7.54± 6.80
		CineMA^{FineTune}	**6.30**± 5.97
BMI	ACDC (SAX)	ResNet^{RandInit}	4.03± 5.30
		CineMA^{RandInit}	4.08± 5.13
		CineMA^{FineTune}	**2.88**± 5.40
Age	M&Ms (SAX)	ResNet^{RandInit}	11.00± 9.28
		CineMA^{RandInit}	11.50± 8.85
		CineMA^{FineTune}	**7.97**± 6.77

Table 11 Landmark localisation performance on the Landmark dataset. 2C, two-chamber. 4C, four-chamber. MAPSE, mitral annular plane systolic excursion. GLS, global longitudinal shortening. MAE, mean absolute error. Values are shown as mean ± std across samples.

Method	View	Model	Euclidean Distance (mm) Valve1	Valve2	Apex	Mean
Heatmap	2C	UNet^{RandInit}	0.85± 0.67	**0.90**± 1.31	**0.93**± 0.65	**0.89**± 0.55
		CineMA^{RandInit}	0.93± 0.84	1.00± 1.37	1.09± 1.16	1.01± 0.72
		CineMA^{FineTune}	**0.83**± 0.70	**0.90**± 1.11	0.98± 0.69	0.90± 0.51
		Xue et al. [8]	2.1± 1.8	2.4± 2.0	2.4± 1.8	2.3± 2.0
	4C	UNet^{RandInit}	**0.97**± 1.08	**1.24**± 1.08	1.07± 1.00	**1.09**± 0.70
		CineMA^{RandInit}	1.12± 1.24	1.32± 1.71	1.27± 1.43	1.24± 0.92
		CineMA^{FineTune}	0.98± 1.07	**1.24**± 1.34	**1.06**± 1.01	**1.09**± 0.76
		Xue et al. [8]	3.4± 2.1	2.1± 1.7	2.8± 1.9	2.77± 2.1
	2C/4C	UNet^{RandInit}	**0.91**± 0.90	**1.07**± 1.21	**1.00**± 0.84	**0.99**± 0.64
		CineMA^{RandInit}	1.03± 1.06	1.16± 1.56	1.18± 1.31	1.12± 0.83
		CineMA^{FineTune}	**0.91**± 0.90	**1.07**± 1.24	1.02± 0.87	1.00± 0.65
Coordinates	2C	ResNet^{RandInit}	1.90± 3.12	1.89± 3.35	1.89± 3.14	1.89± 3.10
		CineMA^{RandInit}	7.94± 3.66	7.90± 3.60	8.35± 4.35	8.06± 3.11
		CineMA^{FineTune}	**1.07**± 0.67	**1.13**± 1.10	**1.08**± 0.72	**1.09**± 0.53
	4C	ResNet^{RandInit}	1.99± 3.58	4.16± 20.54	4.57± 26.45	3.57± 16.76
		CineMA^{RandInit}	5.60± 4.12	5.48± 3.74	5.75± 3.60	5.61± 3.31
		CineMA^{FineTune}	**1.18**± 1.08	**1.39**± 1.01	**1.14**± 0.99	**1.24**± 0.73
	2C/4C	ResNet^{RandInit}	1.94± 3.36	3.02± 14.73	3.23± 18.85	2.73± 12.06
		CineMA^{RandInit}	6.77± 4.07	6.70± 3.86	7.05± 4.20	6.84± 3.43
		CineMA^{FineTune}	**1.12**± 0.90	**1.26**± 1.06	**1.11**± 0.87	**1.16**± 0.64

Method	View	Model	MAE MAPSE (mm)	GLS (%)
Heatmap	2C	UNet^{RandInit}	**0.50**± 0.41	**1.02**± 0.82
		CineMA^{RandInit}	0.57± 0.59	1.12± 1.26
		CineMA^{FineTune}	0.54± 0.51	1.07± 1.00
	4C	UNet^{RandInit}	**0.59**± 0.56	**1.14**± 1.40
		CineMA^{RandInit}	0.67± 0.77	1.31± 1.64
		CineMA^{FineTune}	0.63± 0.59	1.17± 1.34
	2C/4C	UNet^{RandInit}	**0.54**± 0.49	**1.08**± 1.15
		CineMA^{RandInit}	0.62± 0.69	1.21± 1.46
		CineMA^{FineTune}	0.58± 0.55	1.12± 1.18
Coordinates	2C	ResNet^{RandInit}	0.74± 0.78	1.46± 1.34
		CineMA^{RandInit}	4.54± 2.63	5.60± 4.07
		CineMA^{FineTune}	**0.64**± 0.55	**1.15**± 0.99
	4C	ResNet^{RandInit}	0.88± 1.19	1.40± 1.44
		CineMA^{RandInit}	1.73± 1.43	2.61± 2.18
		CineMA^{FineTune}	**0.70**± 0.62	**1.23**± 1.34
	2C/4C	ResNet^{RandInit}	0.81± 1.01	1.43± 1.39
		CineMA^{RandInit}	3.14± 2.54	4.11± 3.59
		CineMA^{FineTune}	**0.67**± 0.59	**1.19**± 1.18

Table 12 Classification performance for systemic diseases and survival prediction on UK Biobank. CineMA was fine-tuned using three random seeds, with the encoder frozen except for the last block. Performance from ViTa [9] was not evaluated on the same dataset, included for reference only. GLM, generalised linear model. XGB, XGBoost. AUROC, area under the receiver operating characteristic curve. AUPRC, area under the precision-recall curve.

Task	AUROC				AUPRC		
	GLM	XGBoost	CineMA^{FineTune}	ViTa	GLM	XGBoost	CineMA^{FineTune}
Hypertension	0.709	0.698	**0.771**	0.746	0.495	0.477	**0.566**
Cancer	0.557	0.525	**0.655**		0.203	0.192	**0.341**
Diabetes	0.709	0.659	**0.871**	0.813	0.064	0.052	**0.305**
1Y Survival	0.590	0.417	**0.727**		0.002	0.001	**0.010**
2Y Survival	0.593	0.516	**0.682**		0.008	0.006	**0.020**
3Y Survival	0.621	0.593	**0.748**		0.020	**0.064**	0.028

Table 13 Segmentation performance on EMIDEC. MYO, myocardium. MI, myocardial infarction. NR, no-reflow. Values are shown as mean ± std across samples.

Model	Dice Score (%)				
	Cavity	MYO	MI	NR	Mean
UNet^{RandInit}	**92.81**± 1.82	81.93± 4.17	36.61± 26.53	68.42± 47.76	69.94± 10.78
CineMA^{RandInit}	75.69± 10.46	51.80± 15.90	7.64± 10.27	54.43± 41.82	47.39± 8.40
CineMA^{FineTune}	92.74± 2.08	**82.43**± 3.75	**38.07**± 23.05	**70.87**± 40.26	**71.03**± 10.58
**Model**	**95% Hausdorff Distance (mm)**				
	Cavity	MYO	Mean		
UNet^{RandInit}	2.95± 1.06	**3.41**± 0.95	21.01± 15.27	**3.18**± 0.97	
CineMA^{RandInit}	10.10± 4.69	11.89± 5.55	31.57± 10.08	10.99± 5.06	
CineMA^{FineTune}	**2.92**± 1.15	3.43± 0.89	**20.39**± 12.10	**3.18**± 0.92	

Table 14 Segmentation performance on MyoPS2020. Values are shown as mean ± std across samples.

Model	Dice Score (%)		
	Scar	Edema Scar	Mean
UNet^{RandInit}	**55.25**± 25.01	58.82± 18.16	**57.04**± 20.55
CineMA^{RandInit}	52.68± 25.17	60.09± 17.58	56.39± 19.83
CineMA^{FineTune}	53.20± 24.20	**60.55**± 16.67	56.88± 19.66

References

[1] Feng, Z.-H., Kittler, J., Awais, M., Huber, P. & Wu, X.-J. Wing loss for robust facial landmark localisation with convolutional neural networks. *2018 IEEE/CVF Conference on Computer Vision and Pattern Recognition* 2235–2245 (2018).

[2] Jacob, A. J. *et al.* Towards a cardiovascular magnetic resonance foundation model for multi-task cardiac image analysis. *Journal of Cardiovascular Magnetic Resonance* **27**, 101967 (2025).

[3] Bernard, O. *et al.* Deep learning techniques for automatic mri cardiac multi-structures segmentation and diagnosis: is the problem solved? *IEEE transactions on medical imaging* **37**, 2514–2525 (2018).

[4] Campello, V. M. *et al.* Multi-centre, multi-vendor and multi-disease cardiac segmentation: the m&ms challenge. *IEEE Transactions on Medical Imaging* **40**, 3543–3554 (2021).

[5] Martín-Isla, C. *et al.* Deep learning segmentation of the right ventricle in cardiac mri: the m&ms challenge. *IEEE Journal of Biomedical and Health Informatics* **27**, 3302–3313 (2023).

[6] Shad, R. *et al.* A generalizable deep learning system for cardiac mri. *Nature Biomedical Engineering* 1–16 (2026).

[7] Bhuva, A. N. *et al.* A multicenter, scan-rescan, human and machine learning cmr study to test generalizability and precision in imaging biomarker analysis. *Circulation: Cardiovascular Imaging* **12**, e009214 (2019).

[8] Xue, H. *et al.* Landmark detection in cardiac mri by using a convolutional neural network. *Radiology: Artificial Intelligence* **3**, e200197 (2021).

[9] Zhang, Y. *et al.* Towards cardiac mri foundation models: Comprehensive visual-tabular representations for whole-heart assessment and beyond. *Medical Image Analysis* **106**, 103756 (2025).
