# Supplementary material for: Development and validation of a versatile foundation model for cine cardiac magnetic resonance image analysis
Source: Commun Med (Lond). 2026 May 13;6:408. doi: 10.1038/s43856-026-01636-0 (PMC13396196; doi:10.1038/s43856-026-01636-0)
Supplement: Supplementary file 1 — Transparent Peer Review File [file 43856_2026_1636_MOESM1_ESM.pdf]

nature portfolio

Peer Review File

Development and validation of a versatile foundation model for cine cardiac magnetic resonance image analysis

Corresponding Author: Mr Yunguan Fu

This file contains all reviewer reports in order by version, followed by all author rebuttals in order by version.

Version 0:

Reviewer comments:

Reviewer #1

(Remarks to the Author)
1. How well does the manuscript justify the choice of the multi-view convolution–transformer masked autoencoder architecture for CineMA compared to purely convolutional or purely transformer-based alternatives? Could the authors clarify why this hybrid approach was chosen and how it balances performance versus computational cost?
2. The model was pre-trained on over 15 million cine CMR images from the UK Biobank. How might the results generalize to populations or imaging protocols not represented in the UK Biobank, and what are the implications for model bias?
3. Were the baseline models (UNet, and ResNet) optimally tuned and retrained under the same conditions? Could the authors provide details on hyperparameter selection, training protocols, and whether baselines were trained using identical subsets and augmentation strategies?
4. The discussion highlights segmentation accuracy, ejection fraction estimation, sensitivity, and specificity. Are these metrics sufficient to capture clinically meaningful differences, or should additional metrics (such as Dice coefficient variance, volumetric consistency, or longitudinal reproducibility) be reported?
5. Eight external datasets were used for evaluation. Can the authors elaborate on the characteristics of these datasets (size, demographics, imaging protocols) and whether any observed performance differences might be attributed to population or acquisition shifts?
6. While CineMA-derived functional metrics were associated with diabetes, cancer, and survival, the model does not predict major adverse cardiovascular events directly. How clinically actionable are these findings, and what are the next steps for integrating CineMA into prognostic models or risk stratification pipelines?
7. The discussion mentions demographic fairness. Could the authors provide more detail on the methods used to assess disparities across subgroups, including statistical tests, effect sizes, and whether any subgroups exhibited systematic bias?
8. CineMA showed limited benefits on LGE and T1 mapping images. Could the authors clarify whether this is due to pre-training limitations, intrinsic modality differences, or architectural constraints, and suggest concrete strategies to extend the model to these modalities?
9. Given the substantial resources required to pre-train foundation models, what strategies do the authors recommend for institutions with limited computational capacity? How feasible is clinical deployment in resource-constrained settings?

Reviewer #2

(Remarks to the Author)
Key results: This paper presents a foundation model for cine cardiac MRI image analysis called CineMA. It is a masked autoencoder with a convolution-transformer architecture that operates on both long-axis and short-axis images. The model was trained on millions of cine images from ~70K subjects in UK Biobank (UKB) using a mean square error loss evaluating its ability to reconstruct masked image patches. It is evaluated in UKB and in 8 additional datasets for a variety of fine-tuning tasks: (1) image segmentation (ventricle, myocardium and scar), (2) ejection fraction quantification (both segmentation-derived and directly regressed), (3) disease classification, (4) landmark localization and associated calculation of long-axis functional measurements and (5) regression of age and BMI and classification of sex and MRI vendor. The accuracy of the fine-tuned foundation model is typically compared with that of two types of models trained from scratch: a UNet or ResNet model as appropriate, or a CineMA model trained from scratch. The foundation model's performance outperforms these baselines or occasionally matches them. The authors also include an investigation into the association of long-axis functional measurements with diabetes, hypertension, cancer and survival in UKB.

Strengths:
- Foundation models are of high utility, and this one is trained on a large dataset and exhaustively validated for a large variety of fine-tuning tasks in many other datasets. I am convinced that it is likely to be an excellent starting point for model training by other researchers with high potential impact and immediate interest.
- The machine learning methods are sound.

Weaknesses:
- The baselines are incomplete. The authors cite three other foundation models that they claim have been insufficiently validated [17-19], including models from their own group. Is it not possible to compare to them? In addition, as you are operating on large public datasets/challenges, are there results from other groups that you can compare to?
- The authors claim that "nnUNet was not included in benchmarks due to its automated optimization pipeline. Fair comparison with such an AutoML optimization to foundation models remains an open research question". I find this unconvincing - readers will want to know if they should use your foundation model or train another model from scratch. Ideally, CineMA would outperform nnUNet as it is so often used by the field, and shown to often outperform alternative UNet setups.
- The authors claim that their model can perform prognostication, but in actuality the CineMA model is used to derive ejection fractions and long-axis functional measurements that are in turn used to train a Cox proportional hazards model. It would be much more interesting if the foundation model's representation itself was used to fine-tune a model for prognosis, as that would much better illustrate the utility of the learned representations for downstream tasks.

Additional comments and questions:
- The statistical reporting in several tables is incomplete, as means are often reported without corresponding uncertainty measures (e.g., standard deviations)
- Is your foundation model applicable to other tasks, e.g., registration, cardiac motion analysis or super-resolution?
- Please explain further why CineMA-FineTune is almost universally worse than the Unet/ResNet models.
- Section 2.8, do you have evidence that a fair model would predict a positive cardiac function outcome with equal probability across ethnic groups? Doesn't this ignore differing outcomes by race? Demographic disparity analysis is also for white vs. non white only. It would be interesting to also evaluate males vs. females, age groups, etc, but perhaps not necessary for this paper.
- The paper is clear and well-written, and the authors do provide enough detail for a researcher to reproduce the work.

Detailed:
- Authors should modulate their claim to be the first cine CMR foundation model, as they cite others [18,19] that were previously developed.
- As many datasets are used, clarity would be improved if all figure and table captions included which dataset was being used.
- Oftentimes, numbers in the main text do not correspond exactly to number in the associated tables. I assume that the numbers in the main text are averages of several experiments from the tables? Please clarify. An example: section 2.5, "specificity (60.06% vs 31.55%) and sensitivity (90.20% vs. 86.84%)", where these numbers are not in the corresponding Table 5.
- Section 2.5: It would be helpful to list the diagnostic labels in the ACDC, M&Ms and M&Ms2 datasets, and to also show the prevalence of these diseases in the datasets.
- Section 2.6: be clear that GLS is never statistically significant
- Please clarify the y-axis in Figure 4b - does 0% "difference in mean" mean that it matches the group not surviving even 1 year? Also, why is there an opposite trend for LVEF vs RVEF?
- Section 4.1: Are the UKB images from instance 2, instance 3, or both?
- Section 5: add citation for the Landmark dataset
- Table 1 caption: add acronym for LGE, T2 and bSSFP
- Table 5: CineMA-RandInit has very low specificity for ACDC SAX and M&Ms SAX?

Typos:
- There are several figures and tables that are never cited in the main text (e.g., Figure 2a, Table 3, Table 9, and perhaps others).
- Page 2: "global systolic functon(e.g., LVEF) AND long-axis functon(e.g., MAPSE and GLS)
- I suspect the y-axis label in Fig 2c top-right "mean ejection fraction (%)" is a typo?
- Section 2.4: "mean errors of 3.72% for LVEF" but all errors in Figure 2c are >5%?
- Figure 3c, order should be MAPSE, GL shortening for consistency
- Consider moving section 4.6 after section 4.7 and tables 10,11 after table 12, to match the flow of the manuscript

Reviewer #3

(Remarks to the Author)
General Assessment
This manuscript presents CineMA, a foundation model for cine cardiac magnetic resonance imaging (CMR), trained on over 15 million images from nearly 75,000 subjects. The authors propose a multi-view convolution–transformer masked autoencoder capable of performing segmentation, diagnosis, and prognostication tasks across multiple independent datasets.
The paper is technically sound, comprehensive, and well-written, with a remarkable scale of pre-training and a robust

validation framework. Its emphasis on generalisability, fairness, and open-source availability makes it a significant advance toward clinically applicable foundation models in cardiovascular imaging.
The authors also thoroughly test the foundation model across several downstream tasks in a fine-tuning setup using diverse datasets, demonstrating robustness and transferability.
However, several clinical contextual elements could be expanded or refined in the discussion to improve completeness and translational impact.

Major Comments
(None: the manuscript is excellent overall. The following are minor but important suggestions to enhance clarity and clinical perspective.)

Minor Comments and Suggestions
1. Clinical Scope – Missing Common Disease Entities
While CineMA is convincingly validated on multiple datasets for segmentation and diagnosis, the discussion omits clinically common entities frequently encountered in CMR:
• Perimyocarditis / Myocarditis: Given the growing clinical importance of inflammatory cardiac diseases, a brief acknowledgment of their absence and potential integration in future datasets would be valuable.
• Valvular disease: Cine imaging is routinely used to assess valvular pathologies such as aortic or mitral regurgitation. Even if such data were unavailable, this could be mentioned as a potential extension for future work.

2. Imaging Modalities – Missing T2-STIR Sequences
The authors note that CineMA was not trained on LGE and T1 mapping sequences, but T2-weighted STIR imaging, which is critical for oedema and inflammation detection, is also absent.
A brief discussion of this omission would be appropriate, clarifying that incorporating T2-STIR or T2 mapping could extend CineMA's clinical utility, especially for myocarditis and acute injury detection.

3. Inter-Reader Variability and Echocardiographic Comparison
The introduction correctly highlights the high inter- and intra-reader variability of manual CMR interpretation.
It would strengthen the discussion to note that echocardiography, which is often used alongside CMR, further contributes to this variability.
CineMA's foundation model approach could introduce a more objective framework for ejection fraction estimation and functional assessment.
Emphasizing this would underline CineMA's potential for clinical reliability and standardization in quantitative cardiac function evaluation.

4. Presentation of Systemic Disease Associations
The manuscript repeatedly highlights associations with systemic diseases (diabetes, hypertension, cancer) across multiple sections, not only in the discussion but also in the abstract and results.
While these findings are interesting, they should be presented with greater caution and positioned as secondary or exploratory analyses, rather than as central outcomes.
A clear statement that these represent correlations rather than causal relationships would improve scientific precision.
Reframing these results as supporting evidence for generalisability, rather than as primary validation findings, would yield a more balanced interpretation.

Summary
This is an outstanding and forward-looking contribution to the field of cardiac imaging AI.
I recommend minor revisions focused on enhancing the clinical discussion by:
• acknowledging missing disease entities (myocarditis, valvular disease),
• mentioning non-utilised but clinically important sequences (T2-STIR),
• emphasizing CineMA's role in mitigating inter-reader variability (including in echocardiography), and
• presenting systemic disease associations more cautiously as secondary exploratory findings.
These revisions will make the manuscript more clinically balanced, precise, and aligned with real-world cardiovascular imaging practice, without requiring additional experimentation.

Version 1:

Reviewer comments:

Reviewer #1

(Remarks to the Author)
Thank you for addressing my comments.

Reviewer #2

(Remarks to the Author)
I would like to thank the authors for addressing my original concerns, and would wholeheartedly recommend this paper for

acceptance.

The authors have created a foundation model using state-of-the-art methods and have performed a comprehensive assessment from a variety of angles that demonstrate its generalizability and where it is sometimes outperformed by other methods (e.g., nnUnet in the case of full training data).

I have a few recommendations for the final manuscript:

- Section 3.4, "CineMAFineTune achieved comparable LVEF prediction accuracy to nnUNet while maintaining a low coefficient of variation (≤ 10.10%), whereas nnUNet’s variation increased to 20.20%". Consider revising this sentence to clarify that this refers to the situation when 10% of training data is available.

- Regarding the hyperparameter selection for the baseline and new models - you are using a fixed number of epochs and a fixed learning rate decay strategy for all tasks and all models. Did you consider using a strategy for learning rate decay and early stopping that is based on validation loss (e.g., keras EarlyStopping, ReduceLROnPlateau and ModelCheckpoint options)? You'd have to choose parameters for patience and learning rate reduction factor to share across all tasks, but this might better automatically adjust the training for each task compared to the fixed parameters you have now but it might be worth considering mentioning in the discussion or for your group's future work.

- Supplemental Table 1: I don't think 'Dice loss + CE loss' was the loss for the heatmap regression?

- In all tables, consider bolding the best results, to highlight the best model for readability

- Results of the Wilcoxon signed-rank test are missing for Figure 2c and perhaps 3c (maybe these ones were all not significant).

- I recommend calculating AUPRC for the results in Figure 4 or indeed anywhere you may have class imbalance (e.g., diabetes prediction).

- For your new Figure 4 results predicting hypertension, cancer, and diabetes, are you predicting incident or prevalent disease? If incident, is it with respect to the imaging date and if so, how do you handle instance 2 vs. instance 3 images?

Reviewer #3

(Remarks to the Author)
The authors have addressed the concerns raised in my previous review in a clear and satisfactory manner. The revisions have improved the methodological transparency and clarity of the manuscript. I have no further comments.

Version 2:

Reviewer comments:

Reviewer #2

(Remarks to the Author)
Thank you for addressing my final comments. This is a fantastic paper.

Absolutely no need to send the paper back to me for re-review, but a few small fixes that the authors can put into the final proof:
- AUPRC and AUROC are typically reported between 0-1 and not as percentages.
- The authors added the UKB fields for blood pressure and cancer but not diabetes.

And a comment:
- The field used for blood pressure (2966) is self-reported data only and subject to participant recall. In future the authors may want to consider ICD codes for hypertension as well.

Open Access This Peer Review File is licensed under a Creative Commons Attribution 4.0 International License, which permits use, sharing, adaptation, distribution and reproduction in any medium or format, as long as you give appropriate credit to the original author(s) and the source, provide a link to the Creative Commons license, and indicate if changes were made.
In cases where reviewers are anonymous, credit should be given to 'Anonymous Referee' and the source.
The images or other third party material in this Peer Review File are included in the article's Creative Commons license, unless indicated otherwise in a credit line to the material. If material is not included in the article's Creative Commons license and your intended use is not permitted by statutory regulation or exceeds the permitted use, you will need to obtain permission directly from the copyright holder.
To view a copy of this license, visit https://creativecommons.org/licenses/by/4.0/

Dear Editor,

Thank you for the opportunity to revise our manuscript, "Development and validation of a versatile foundation model for cine cardiac magnetic resonance image analysis tasks." We are grateful to the reviewers for their constructive feedback, which has substantially strengthened the work.

We have performed a major revision addressing all reviewer comments. The key changes include:

1. Add nnUNet as a strong baseline for segmentation, demonstrating comparable accuracy and superior consistency of CineMA, as suggested by Reviewer 2
2. Replace the indirect prognostication pipeline with end-to-end fine-tuned CineMA models that predict systemic diseases and survival directly from images, as suggested by Reviewer 2
3. Add architectural ablation studies justifying the hybrid convolution–transformer design, as suggested by Reviewer 1
4. Expanding the fairness analysis across sex, race, BMI, and age subgroups, as suggested by Reviewer 2
5. Add dataset characteristics and standard deviations across all tables, as suggested by Reviewer 1 and 2

A point-by-point response to all reviewer comments follows below, with revised manuscript text shown in italics and corresponding Action statements specifying where changes were made.

Best regards,
Yunguan Fu

Referee expertise:

Referee #1: Cardiac MR, Pediatrics, MR physics

Referee #2: Computer science, Deep learning for cardiac MR, Medical image analysis

Referee #3: Radiology, Medical image analysis, Deep learning for cardiac MR

Reviewers' comments:
Reviewer #1 (Remarks to the Author):

1. How well does the manuscript justify the choice of the multi-view convolution–transformer masked autoencoder architecture for CineMA compared to purely convolutional or purely transformer-based alternatives? Could the authors clarify why

this hybrid approach was chosen and how it balances performance versus computational cost?
Response: The choice of a multi-view convolution–transformer hybrid for CineMA was driven by three specific considerations: computational efficiency within the MAE framework, the geometric flexibility required for multi-view fusion, and the performance gains provided by local inductive biases in convolutional layers.

First, following previous work (Zhou et al. 2023, Nature), we use masked auto-encoder (MAE) as unsupervised pre-training framework. The model leverages a sparse processing mechanism where only visible image tokens are processed by the Transformer encoder. This provides a significant computational advantage over purely convolutional architectures, which are inherently dense and would require processing the entire image volume, including masked regions, thereby increasing the computational overhead.

Second, an objective of this work is to develop a single, unified foundation model capable of processing all standard views, rather than maintaining disparate models for 3D short-axis (SAX) and 2D long-axis (LAX) acquisitions. We therefore follow the MultiMAE framework (Bachmann et al., 2022), we encode each view separately and concatenate these tokens in latent space, learning cross-view anatomical dependencies through self-attention.

Third, the hybrid architecture has been reported to yield superior representation learning performance, as it combines the advantages of convolutational networks (capturing fine-grained local features) and Transformer-based models (modelling long-range dependencies), as demonstrated in ConvMAE (Gao et al., 2022). We added an ablation study for segmentation task on ACDC, M&Ms, and M&Ms2 datasets (Supplementary Table 2). When trained from randomly initialised weights, the hybrid architecture (CineMA) constantly obtained higher mean Dice score then variant models without two convolutional layers. Particularly, when removing the convolutional layers, we have to increase the patch size by 4x, as keeping the same patch size increased the number of patches which slows down the training by more than 10x, empirically. These results demonstrate that the initial convolutional stages are necessary to capture the fine-grained anatomical features.

Revision: We have added the ablation in Supp. Table 2 and updated the method section 2.4 and results section 3.1.

Table 2 Ablation of segmentation model architecture on ACDC, M&Ms, and M&Ms2 datasets. All models were trained from randomly initialised weights. RV, right ventricle. MYO, myocardium. LV, left ventricle. Values are shown as mean ± std across samples.

Dataset	Model	Patch size	Dice Score (%) RV	MYO	LV	Mean
ACDC	w/ Conv	4	90.12± 6.28	86.52± 7.56	92.08± 9.03	89.57± 6.08
	w/o Conv	16	85.66± 9.06	81.45± 5.65	89.55± 8.09	85.55± 5.49
M&Ms	w/ Conv	4	84.70± 10.07	81.16± 6.96	89.24± 7.66	85.03± 6.49
	w/o Conv	16	80.58± 14.38	75.47± 8.19	85.76± 10.05	80.60± 8.88
M&Ms2	w/ Conv	4	84.97± 14.04	81.92± 9.08	91.00± 9.63	85.96± 9.48
	w/o Conv	16	81.44± 15.55	76.79± 9.73	88.58± 10.30	82.27± 10.29
**Dataset**	**Model**	**Patch size**	**95% Hausdorff Distance (mm) RV**	**MYO**	**LV**	**Mean**
ACDC	w/ Conv	4	5.43± 5.20	4.60± 7.03	4.88± 9.25	4.99± 5.82
	w/o Conv	16	7.87± 6.05	5.42± 4.23	5.81± 4.88	6.37± 4.15
M&Ms	w/ Conv	4	10.25± 13.86	6.24± 4.67	6.80± 5.33	7.76± 6.05
	w/o Conv	16	10.33± 8.85	8.44± 6.82	9.36± 8.59	9.38± 6.70
M&Ms2	w/ Conv	4	14.00± 20.00	7.41± 6.19	7.41± 6.38	9.66± 8.47
	w/o Conv	16	12.89± 12.94	8.78± 8.21	8.74± 8.80	10.04± 7.75

With convolutional layers in CineMA, the effective patch size before the transformer is 16x16 for SAX slices. For a transformer-only model without convolutional layers, the patch size before the transformer would be 4x4, increasing the number of tokens16-fold, which slows training by more than 10 times, rendering both pre-training and downstream fine-tuning infeasible. Therefore, a transformer-only model is constructed by removing convolutional layers and increase patch size from 4x4 to 16x16.

Following ConvMAE [24], we incorporated convolutional layers into the encoding and decoding stages to capture high-resolution local features while effectively reducing the token count. This hybrid approach provides a performance advantage (Supplementary Table 2) over purely transformer-based architectures with larger patch sizes and substantially accelerates training compared to transformers with small patch sizes. [...] Furthermore, on segmentation tasks, we compared the proposed conv-transformer architecture with a transformer-only model~(Supplementary Table 2) and observed a drop of mean Dice score by 3.69% to 4.43% across all datasets (ACDC, M&Ms, and M&Ms2). We therefore excluded transformer-only models from further comparisons.

2. The model was pre-trained on over 15 million cine CMR images from the UK Biobank. How might the results generalize to populations or imaging protocols not represented in the UK Biobank, and what are the implications for model bias?

Response: To understand the potential biases inherent in the UK Biobank (UKB) cohort, we designed our downstream evaluation suite to rely heavily on external, non-UKB datasets. All datasets used in segmentation, classification, regression and long-axis function prediction are non-UKB data. These datasets comprise various imaging protocols, with scanner manufacturers and data sources (by country) specified in Table 1. We have also added the distribution of age, height, weight, and body-mass index on UKB, ACDC, and M&Ms datasets when available in Supplementary Figure 1. For instance,

the individuals in UKB have minimum 45 years old, with an average age of 65.8, while >30% individuals in M&Ms are younger than 45.

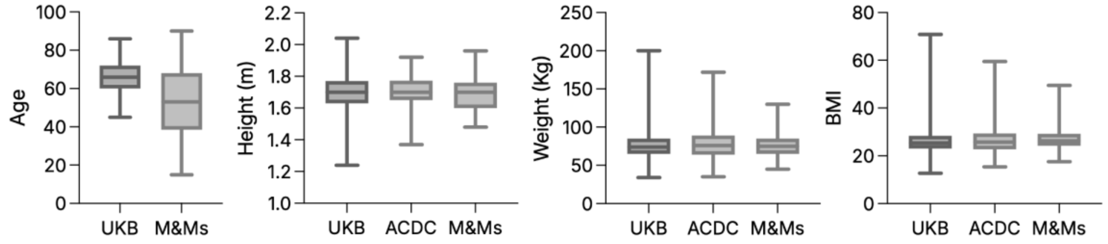

Fig. 1 Demographic distribution between UK Biobank (UKB) data, ACDC and M&Ms datasets on available labels. BMI, body mass index.

The robustness of CineMA against domain and population shifts is evidenced by the evaluation on Kaggle and Rescan datasets. Specifically, segmentation models were fine-tuned on the ACDC and M&Ms datasets and subsequently tested on the Kaggle and Rescan datasets. This ensures that the model is evaluated on images entirely unseen during both pre-training and fine-tuning. As shown in Figure 2b in the paper, the Kaggle/Rescan datasets exhibit a 20% distributional shift in left ventricular ejection fraction (LVEF) compared to the pre-training data. Especially, the fine-tuned models maintained accurate LVEF prediction with high consistency (coefficient of variation <10.1%) on the Rescan dataset even when only using 10% of training data. These results indicate that the learned representation from UKB does generalise to other imaging protocols and individual distributions.

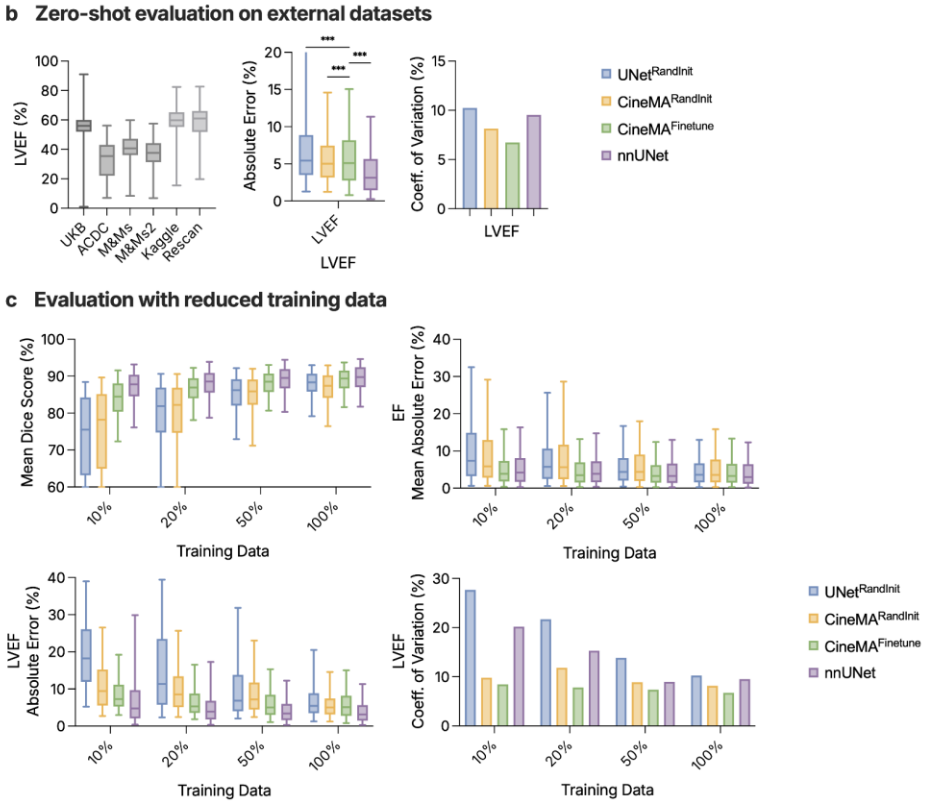

The sensitivity of model performance to these demographic and acquisition factors has been further quantified in section 3.7, when we finetune the models to predict systemic diseases and survivals. These observations can be utilised to guide further generalisation to unseen clinical populations.

Revision: We updated result section 3.2, 3.4, and 3.7.

These cohorts present a domain shift from the UKB pre-training data, encompassing diverse demographic distributions (Supplementary Fig. 1), heterogeneous imaging protocols (Table 1), and a wide spectrum of cardiovascular pathologies. Consequently, they provide an important benchmark for assessing the model's generalisability to clinical populations and technical parameters not represented in the initial pre-training.

CineMA^{FineTune} achieved comparable LVEF prediction accuracy to nnUNet while maintaining a low coefficient of variation (≤10.10%), whereas nnUNet's variation increased to 20.20%. These results suggest that although nnUNet's dataset-specific tuning strategy improves performance on similar data distributions, it may be less robust to distribution shifts. In contrast, pre-training regularises CineMA's representation learning during fine-tuning, yielding better generalisation capacity, particularly in low-data settings.

We further assessed whether the model's accuracy varies across demographic subgroups (sex, race, BMI, and age). As shown in Figure 4, the fine-tuned CineMA model demonstrates comparable performance across sex, race, and BMI groups when predicting systemic diseases. Notably, despite UK Biobank data predominantly consisting of White subjects (90.86%), we did not observe significant performance deterioration in non-White groups, despite the class imbalances (Table 3). For groups with different ages, we observed changes in performance between groups, indicating potential room for investigation in future studies.

3. Were the baseline models (UNet, and ResNet) optimally tuned and retrained under the same conditions? Could the authors provide details on hyperparameter selection, training protocols, and whether baselines were trained using identical subsets and augmentation strategies?

Response: The baseline models were trained on exactly same training protocols, with identical training, validation, and test splits to ensure fair comparisons. The hyperparameters, and data augmentation strategies are summarised in Supplementary Table 1. We have also open-sourced the code for reproducible research. These hyperparameters were selected empirically without tuning. We trained each model three times with distinct random seeds to reduce the impact of training randomness. Tuning all models manually across all tasks was not practical in the evaluation. We therefore have added nnUNet as a strong baseline model for segmentation task, where the hyperparameters were automatically tuned per dataset. We showed that without hyperparameter tuning, the proposed CineMA model approaches nnUNet performance (Figure 2), while having lower coefficient of variation on the unseen Rescan datasets.

Table 1 Hyperparameters for downstream task model training. If the training dataset size is smaller than the preset batch size, the batch size is reduced to the closest power of two. CE, cross-entropy. MSE, mean squared error. MCC, Matthews correlation coefficient. ROC AUC, area under the receiver operating characteristic curve.

Hyperparameter	Classification	Regression	Segmentation
Total Epochs	800	800	4000
Warmup Epochs	10	10	50
Peak Learning Rate	0.001	0.001	0.001
End Learning Rate	10^{-5}	10^{-5}	10^{-5}
Batch Size	64	64	64
Loss	CE Loss	MSE	Dice Loss + CE Loss
Augmentation	Contrast adjustment, Gaussian noise, Affine transformation.	Contrast adjustment, Gaussian noise, Affine transformation.	Contrast adjustment, Gaussian noise, Affine transformation, Crop dropout.
Validation Frequency	20	20	100
Validation Patience	5	5	5
Validation Metric	MCC	Absolute Error	Dice Score
Hyperparameter	Landmark localisation		Systemic disease
	Heatmap	Coordinate	Survival
Total Epochs	400	400	20
Warmup Epochs	10	10	2
Peak Learning Rate	0.001	0.001	0.001
End Learning Rate	10^{-5}	10^{-5}	10^{-5}
Batch Size	64	64	256
Loss	Dice Loss + CE Loss	Wing Loss [1]	Focal Loss
Augmentation	Contrast adjustment, Gaussian noise, Affine transformation.	Contrast adjustment, Gaussian noise, Affine transformation.	Contrast adjustment, Gaussian noise, Affine transformation.
Validation Frequency	20	20	1
Validation Patience	5	5	5
Validation Metric	L^2 distance	L^2 distance	ROC AUC

Revision: We have updated the method section 2.5.

*For each task, all models were trained with same training protocols, identical training, validation, and test splits to ensure fair comparisons. For all models except nnUNet, the same set of hyperparameters were used without exhaustive search (*Supplementary Table 1*).*

4. The discussion highlights segmentation accuracy, ejection fraction estimation, sensitivity, and specificity. Are these metrics sufficient to capture clinically meaningful differences, or should additional metrics (such as Dice coefficient variance, volumetric consistency, or longitudinal reproducibility) be reported?

Response: We agree that while Dice scores and diagnostic accuracy are common evaluation metrics in cardiac image analysis, more evaluations are required to represent clinical relevance. We have added the mean absolute errors for several metrics of clinical importance, including the LV volume, myocardial volume, RV volume and the ejection fraction (EF) as shown in Supplementary Table 5 and 7.

Table 5 Ventricle and myocardium segmentation performance in volume prediction. SAX dataset represents the combined test sets from ACDC, M&Ms, and M&Ms2 on SAX views. SAX, short-axis. RV, right ventricle. MYO, myocardium. LV, left ventricle. MAE, mean absolute error. Values are shown as mean ± std across samples.

Dataset	Model	Volume MAE (ml)			
		RV	MYO	LV	Mean
ACDC (SAX)	$\text{UNet}^{\text{RandInit}}$	11.25± 11.27	8.14± 7.08	5.78± 4.67	8.39± 5.41
	$\text{CineMA}^{\text{RandInit}}$	8.81± 9.92	8.24± 11.42	6.16± 4.86	7.74± 6.04
	$\text{CineMA}^{\text{FineTune}}$	9.19± 10.34	7.59± 5.75	5.06± 3.59	7.28± 4.27
	nnUNet	8.35± 10.54	7.11± 5.83	5.46± 4.27	6.98± 4.46
M&Ms (SAX)	$\text{UNet}^{\text{RandInit}}$	10.46± 9.10	13.61± 11.82	9.42± 8.90	11.16± 6.73
	$\text{CineMA}^{\text{RandInit}}$	12.64± 10.89	12.83± 9.45	8.77± 7.27	11.41± 5.95
	$\text{CineMA}^{\text{FineTune}}$	9.24± 9.16	11.47± 8.86	8.50± 7.44	9.74± 5.61
	nnUNet	10.10± 10.03	12.81± 12.38	8.98± 8.52	10.63± 7.24
M&Ms2 (SAX)	$\text{UNet}^{\text{RandInit}}$	12.61± 11.55	8.14± 6.57	8.17± 7.36	9.64± 5.68
	$\text{CineMA}^{\text{RandInit}}$	17.38± 22.10	8.61± 7.27	9.03± 7.33	11.67± 9.14
	$\text{CineMA}^{\text{FineTune}}$	12.45± 13.24	7.31± 6.13	7.46± 6.34	9.08± 5.71
	nnUNet	9.87± 10.00	7.53± 6.29	7.25± 7.08	8.21± 5.09
	M&Ms2 [5]	[8.89, 9.79]			
SAX	$\text{UNet}^{\text{RandInit}}$	11.57± 10.64	10.29± 9.44	8.31± 7.78	10.06± 6.15
	$\text{CineMA}^{\text{RandInit}}$	14.26± 17.18	10.22± 9.10	8.51± 7.06	10.99± 7.71
	$\text{CineMA}^{\text{FineTune}}$	10.71± 11.48	8.99± 7.54	7.52± 6.58	9.07± 5.53
	nnUNet	9.74± 10.09	9.54± 9.49	7.67± 7.46	8.98± 6.11

Table 7 Zero-shot performance of segmentation models on left ventricular ejection fraction regression using short-axis images. Models were trained on ACDC, M&Ms, and M&Ms2, respectively, then evaluated on Kaggle and Rescan. When the training set includes all three datasets, statistics are calculated across nine models with different training datasets and seeds. The last four rows correspond to a combined test set from Kaggle and Rescan. Vol MAE, average mean absolute error for end-diastolic and end-systolic volumes. EF, ejection fraction. MAE, mean absolute error. CV, coefficient of variation. Values are shown as mean ± std across samples.

Test	Train	Model	Vol MAE (ml)	EF MAE (%)	EF CV (%)
Kaggle	ACDC	UNet^{RandInit}	16.88 ± 19.17	6.24 ± 5.69	
		CineMA^{RandInit}	13.65 ± 15.89	5.98 ± 6.61	
		CineMA^{FineTune}	13.68 ± 16.37	5.21 ± 4.00	
		nnUNet	13.08 ± 18.52	5.35 ± 5.82	
	M&Ms	UNet^{RandInit}	16.29 ± 17.47	5.05 ± 6.13	
		CineMA^{RandInit}	15.99 ± 18.09	5.90 ± 7.07	
		CineMA^{FineTune}	15.03 ± 17.38	3.83 ± 3.59	
		nnUNet	13.96 ± 15.93	4.00 ± 4.18	
	M&Ms2	UNet^{RandInit}	11.36 ± 14.61	4.15 ± 5.33	
		CineMA^{RandInit}	12.53 ± 16.58	5.32 ± 7.35	
		CineMA^{FineTune}	11.67 ± 15.47	3.77 ± 4.03	
		nnUNet	11.29 ± 15.57	3.70 ± 4.52	
	ACDC M&Ms M&Ms2	UNet^{RandInit}	14.85 ± 17.39	5.15 ± 5.79	
		CineMA^{RandInit}	14.06 ± 16.93	5.74 ± 7.02	
		CineMA^{FineTune}	13.46 ± 16.51	4.27 ± 3.93	
		nnUNet	12.78 ± 16.75	4.35 ± 4.94	
	UKB	Shad et al. [6]		6.88	
Rescan	ACDC	UNet^{RandInit}	17.53 ± 21.30	7.69 ± 8.01	17.42
		CineMA^{RandInit}	24.29 ± 23.97	7.18 ± 5.78	10.26
		CineMA^{FineTune}	34.58 ± 23.39	8.43 ± 6.25	9.79
		nnUNet	17.17 ± 14.95	3.89 ± 5.61	12.67
	M&Ms	UNet^{RandInit}	9.50 ± 8.40	3.62 ± 3.87	6.87
		CineMA^{RandInit}	11.09 ± 10.37	4.04 ± 3.92	6.71
		CineMA^{FineTune}	10.09 ± 7.98	3.98 ± 3.91	7.39
		nnUNet	11.35 ± 5.75	4.33 ± 3.96	8.02
	M&Ms2	UNet^{RandInit}	18.12 ± 10.31	4.43 ± 4.23	8.50
		CineMA^{RandInit}	21.89 ± 21.13	4.66 ± 5.47	10.44
		CineMA^{FineTune}	17.58 ± 9.54	3.95 ± 3.65	6.51
		nnUNet	20.87 ± 8.02	4.41 ± 3.39	6.92
	ACDC M&Ms M&Ms2	UNet^{RandInit}	13.04 ± 11.13	4.80 ± 5.12	10.24
		CineMA^{RandInit}	18.35 ± 16.24	5.13 ± 4.81	8.14
		CineMA^{FineTune}	20.19 ± 11.09	5.22 ± 4.03	6.73
		nnUNet	16.46 ± 11.05	4.21 ± 4.42	9.53
	Rescan	Bhuva et al. [7]	9 ± 9	4 ± 4	8.8
Kaggle Rescan	ACDC M&Ms M&Ms2	UNet	15.23 ± 16.26	5.46 ± 5.68	
		CineMA^{RandInit}	15.96 ± 18.23	5.65 ± 6.37	
		CineMA^{FineTune}	16.07 ± 16.97	4.65 ± 4.07	
		nnUNet	14.03 ± 15.16	4.30 ± 4.77	

While the evaluation is constrained by the available labels in the public datasets, we address clinical relevance by prioritising domain generalisation and longitudinal reproducibility. To assess robustness under real-world clinical heterogeneity, we evaluated the model on Kaggle and Rescan datasets, representing distributions entirely unseen during pre-training and fine-tuning. Specifically, the mean absolute error for left ventricular (LV) volume increased from a range of 7–9 ml on fine-tuning data test splits to 12–25 ml on the unseen external datasets. However, the error in predicting the Left Ventricular Ejection Fraction (LVEF) remained stable within a 3–4% range. This difference suggests that volume estimations are potentially more sensitive to labelling biases than ejection fractions.

Moreover, on Rescan dataset, which includes two scans per individual within one week, we reported the coefficient of variation (CV) in Dice score to assess the longitudinal reproducibility, a clinically important measure for ensuring reliable patient monitoring across serial examinations. CineMA^{FineTune} maintains a low coefficient of variation

(≤10.10%) when using 10%, 20%, 50%, and 100% of the available training data. In contrast, nnUNet's variation increased from 9.53% to 20.20% when restricted to 10% training data. This low-data resilience is particularly relevant for real-world clinical deployment, where labelled datasets are often scarce and resource-constrained institutions may not have the capacity to curate large annotated cohorts.

Revision: We included these results and discussions in section 3.3 and 3.4

Across fine-tuned variants, CineMA^{FineTune} achieved a CV of 6.73% across paired scans, significantly lower than nnUNet (9.53%) and other baselines. Notably, despite not being trained on the Rescan dataset, CineMA^{FineTune} also outperformed the previously reported CV of 8.8% by Bhuva et al. [6]. This reduction in variability suggests that pre-training acts as a robust regulariser, an advantage that becomes more pronounced in low-data settings, as examined in the next section.

CineMA^{FineTune} achieved comparable LVEF prediction accuracy to nnUNet while maintaining a low coefficient of variation (≤10.10%), whereas nnUNet's variation increased to 20.20%. These results suggest that although nnUNet's dataset-specific tuning strategy improves performance on similar data distributions, it may be less robust to distribution shifts. In contrast, pre-training regularises CineMA's representation learning during fine-tuning, yielding better generalisation capacity, particularly in low-data settings. This consistency across heterogeneous acquisitions addresses a key clinical need: reducing the operator- and scanner-dependent variability that currently limits CMR interpretation, thereby supporting more standardised cardiac function assessment in multi-centre studies and longitudinal monitoring. Consequently, CineMA can be considered a viable alternative to nnUNet for clinical studies, reducing the manual annotation burden while preserving generalisation across populations.

5. Eight external datasets were used for evaluation. Can the authors elaborate on the characteristics of these datasets (size, demographics, imaging protocols) and whether any observed performance differences might be attributed to population or acquisition shifts?

Response: We have added the information of scanners and source country of data in Table 1, which also includes the number of images used. We additionally visualised the distribution of age, height, weight and BMI in these datasets in Supplementary Figure 1.

Importantly, CineMA's improvements over baselines were observed consistently across all evaluated datasets, despite their heterogeneous characteristics including different scanner vendors (Siemens, Philips, GE, Canon), field strengths (1.5T and 3T), countries (UK, France, Spain, Germany, Canada, US, China), and pathology distributions (Table 2). The consistent improvements across these diverse settings suggest that CineMA's benefits are not limited to specific populations or imaging protocols. We also studied the impact of training data for segmentation tasks as shown in Fig. 2c. Overall, more training

data improves all models' prediction accuracy while fine-tuned models have a better consistency.

Revision: we have added Supplementary Figure 1 and updated table 1.

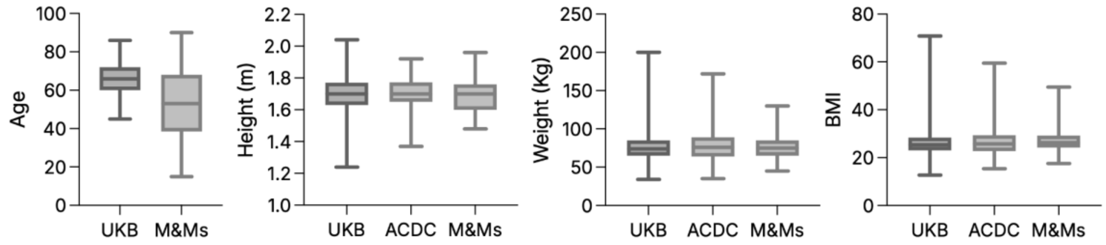

Fig. 1 Demographic distribution between UK Biobank (UKB) data, ACDC and M&Ms datasets on available labels. BMI, body mass index.

Table 1: Datasets used in pre-training and downstream tasks. n is the number of subjects in each dataset. Each dataset includes different views and may contain a different number of frames. SAX, short-axis. LAX, long-axis. nC, n-chamber. EF, ejection fraction. BMI, body mass index. LGE, late gadolinium enhancement. bSSFP, balanced steady-state free precession.

Dataset	Modality	Views	n	Tasks	Scanner	Country
UKB [23]	Cine	SAX; LAX 2C/3C/4C	74,916	Pre-training; Diabetes, hypertension, cancer; 1-/2-/3-year survival.	1.5T Siemens Aera.	UK
ACDC [7]	Cine	SAX	150	Ventricle segmentation; EF and BMI regression; Disease classification.	1.5T Siemens Aera, 3T Siemens Trio Tim.	France
M&Ms [8]	Cine	SAX	375	Ventricle segmentation; EF and age regression; Disease and sex classification.	1.5T Siemens Avanto, 1.5T Philips Achieva, 1.5T GE Signa Excite, 1.5T Canon Vantage Orian, 3T Siemens Skyra.	Spain, Canada, Germany
M&Ms2 [9]	Cine	SAX; LAX 4C	360	Ventricle segmentation; EF regression; Disease and vendor classification.	1.5T Philips Achieva, 1.5T GE Signa Excite/HDxt/Explorer, 1.5T Siemens Avanto (Fit), 1.5T Siemens Symphony (Tim), 3T GE Signa HDxt, 3T Siemens TrioTim.	Spain
Kaggle [10]	Cine	SAX	440	EF regression.	N/A	US
Rescan [6]	Cine	SAX	206	EF regression.	1.5T, 3T Siemens, Philips Aera, Achieva, Avanto.	UK
Landmark [11]	Cine	LAX 2C/4C	1,444	Landmark localisation; Long-axis function regression.	1.5T Siemens Aera, 3T Siemens Magnetom Prisma	UK
EMIDEC [12]	LGE	SAX	100	Ventricle segmentation.	1.5T/3T Siemens	France
Myops2020 [13]	LGE, T2, bSSFP	SAX	25	Scar segmentation.	1.5T Philips Achieva	China

6. While CineMA-derived functional metrics were associated with diabetes, cancer, and survival, the model does not predict major adverse cardiovascular events directly. How clinically actionable are these findings, and what are the next steps for integrating CineMA into prognostic models or risk stratification pipelines?

Response: We have updated the studies on systemic diseases and survivals: we have fine-tuned the foundation model to predict hypertension, cancer, diabetes, and 1/2/3 year survival, separately. The results have been summarised in the updated Figure 4, where the fine-tuned models have surpassed the baseline machine learning models that were trained on the same datasets, but only using CineMA derived functional metrics. These comparisons demonstrate that, the pre-trained foundation models can be finetuned to predict events directly from cardiac CMR images.

We agree with the reviewer that integrating these capabilities into specific clinical risk stratification pipelines (e.g., for major adverse cardiovascular events) represents an important translational goal, as it does for all imaging biomarkers. However, we would argue that the ability to demonstrate predictive capacity for a range of systemic conditions, as tested in this study, is arguably a more challenging endeavour. These results provide evidence that CineMA captures biologically meaningful cardiac signatures, which is a necessary foundation before building targeted clinical risk stratification tools. For instance, the studies of systemic diseases and survival were only limited to UKB dataset, the next steps should involve validating these across other external datasets where labels are available. Furthermore, CineMA-derived functional metrics and predictions of diseases and survivals can be integrated together with non-imaging clinical data in multimodal fusion to improve existing models and offer

Revision: We have updated the discussion section to include these insights.

First, although CineMA was evaluated on non-cardiac conditions and survival prediction, these analyses were limited to UK Biobank data and did not include cardiac clinical outcomes such as major adverse cardiovascular events. Future work should evaluate outcome-prediction models on additional clinical endpoints and external cohorts to determine the added value of CineMA for improving prognostic models and risk stratification pipelines.

Finally, for population-level studies, CineMA models enable high-throughput derivation of digital biomarkers, including cardiac functional indices and estimates of cardiac and systemic disease. When integrated into prognostic models or risk stratification pipelines, these CineMA-derived metrics facilitate complex multi-modal association studies while providing image-based interpretability for downstream clinical predictions.

7. The discussion mentions demographic fairness. Could the authors provide more detail on the methods used to assess disparities across subgroups, including statistical tests, effect sizes, and whether any subgroups exhibited systematic bias?

Response: Since we have fine-tuned CineMA to predict systemic diseases and survivals, we updated the fairness analysis to report the model’s ROC AUC on different groups.

Revision: we updated figure 4, section 3.7.

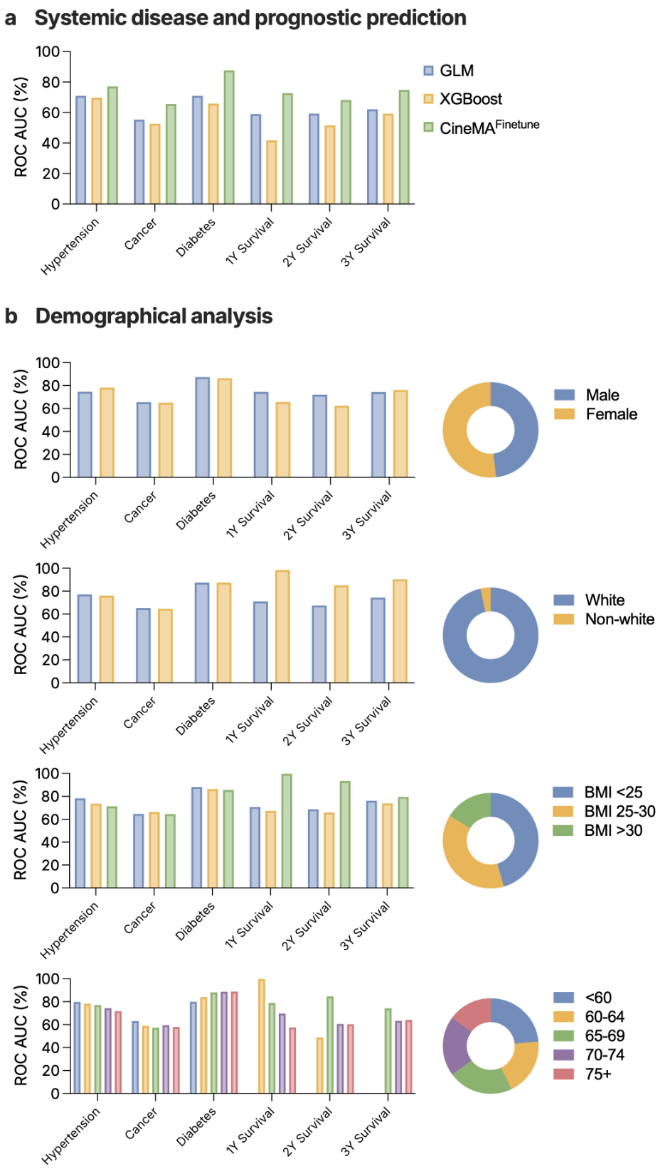

We further assessed whether the model's accuracy varies across demographic subgroups (sex, race, BMI, and age). As shown in Figure 4, the fine-tuned CineMA model demonstrates comparable performance across sex, race, and BMI groups when predicting systemic diseases. Notably, despite UK Biobank data predominantly consisting of White subjects (90.86%), we did not observe significant performance deterioration in non-White groups, despite the class imbalances (Table 3). For groups with different ages, we observed changes in performance between groups, indicating potential room for investigation in future studies.

8. CineMA showed limited benefits on LGE and T1 mapping images. Could the authors clarify whether this is due to pre-training limitations, intrinsic modality differences, or architectural constraints, and suggest concrete strategies to extend the model to these modalities?

Response: On the EMIDEC dataset, the fine-tuned model improved significantly from the randomly initialized version (mean Dice 47.39% to 71.03%), performing comparably to the UNet baseline (69.94%). We hypothesize that the inductive bias in convolutional networks provides specific architectural advantages in this context. Conversely, on the Myops2020 dataset, the improvement was limited (56.88% to 56.39%, comparable to UNet 57.04%), which we attribute to the lack of LGE/T1 images during pre-training given the small sample size (n=20).

To extend the model to these modalities, we have extended the discussion section to suggest that future work should develop modality-specific foundation models or perform continued unsupervised pre-training on LGE/T1 images and the implementation of modality-specific adapters to bridge the distributional gap.

Revision: we have updated the discussions.

In addition, we observed limited benefits of CineMA on LGE, T1, and T2 mapping images (Supplementary Table 13, Supplementary Table 14), which is not surprising given that these modalities were not included in pre-training. T2-weighted imaging, including T2-STIR sequences critical for detecting myocardial oedema in myocarditis and acute cardiac injury, was only available in the limited MyoPS2020 dataset (n=25). Furthermore, the evaluation datasets did not include myocarditis or other inflammatory cardiomyopathies, nor valvular pathologies such as aortic or mitral regurgitation, which are commonly assessed using cine CMR in clinical practice. Future work should develop modality-specific foundation models [31] or perform continued unsupervised pre-training on LGE/T1 images and the implementation of modality-specific adapters to bridge the distributional gap.

9. Given the substantial resources required to pre-train foundation models, what strategies do the authors recommend for institutions with limited computational capacity? How feasible is clinical deployment in resource-constrained settings?

Response: The concern regarding the "compute gap" in foundation model development is central to the democratisation of AI in healthcare. Rather than requiring massive infrastructure, our findings suggest that CineMA offers a viable path forward for resource-constrained institutions through transfer learning.

The evidence presented in Figure 2c is informative. When labelled data is scarce, CineMA's pre-trained weights provide a meaningful initialisation advantage, providing nnUNet-level segmentation accuracy and reduced variation across longitudinal studies. Fine-tuning CineMA would not require large computational costs. Furthermore, as the studies performed on systemic disease prediction, we can fine-tune only part of the models, enabling using large scale datasets within reasonable compute budgets.

Regarding clinical deployment, the feasibility in constrained environments is supported by the model's lean architectural footprint. Because CineMA operates with hundreds of millions of parameters, rather than the billions seen in contemporary large language models, it maintains a highly favourable memory-to-performance ratio. During inference

on a single patient case, the memory demand drops below 1 GB, moving the model out of the realm of specialised data centres and into that of standard clinical workstations or consumer-grade hardware already present in many medical facilities.

Furthermore, the released comprehensive codebase with diverse use cases serves as a practical bridge for this transition, lowering the technical overhead for integration and ensuring that the primary challenge for a clinic is no longer "how to afford the compute" but rather "how to best apply the model" to their specific patient population. Ultimately, our findings support a shift in the AI paradigm: moving away from centralized, resource-heavy systems toward a distributed model where high-performance clinical tools are accessible at the edge.

Revision: We have updated the discussion section.

To facilitate clinical translation and lower the computational barriers to foundation model development, we have released the source code and weights for all pre-trained and fine-tuned CineMA models. For practical implementation, we propose a stratified deployment strategy. Practitioners should prioritise direct inference for short-axis segmentation using the M&Ms-2 fine-tuned model, which was trained on the largest dataset and demonstrates the best performance on unseen data distributions. Prediction stability can be further enhanced through model ensembling across different training seeds and datasets. Especially, these segmentation models can be used for estimating ventricle volumes in order to label end-diastolic and end-systolic frames, which could help reduce model training data. In scenarios where local labels are available, CineMA serves as a robust alternative to nnUNet, particularly in low-data regimes where pre-training provides a regularisation effect. For disease detection and classification, the released weights enable immediate inference when the target pathologies align with existing cohorts. Fine-tuning the CineMA backbone is recommended over standard ResNet architectures for novel conditions. For long-axis functional estimation, the provided weights allow initial zero-shot validation on custom cohorts prior to adaptation. For downstream tasks not evaluated in this study, such as image registration or super-resolution, the pre-trained CineMA encoders can function as optimised feature extractors, as their latent embeddings capture complex cardiac geometry that can be effectively transferred to predict velocity fields or recover sub-pixel anatomical details[33]. Finally, for population-level studies, CineMA models enable high-throughput derivation of digital biomarkers, including cardiac functional indices and estimates of cardiac and systemic disease. When integrated into prognostic models or risk stratification pipelines, these CineMA-derived metrics facilitate complex multi-modal association studies while providing image-based interpretability for downstream clinical predictions.

Reviewer #2 (Remarks to the Author):

Key results: This paper presents a foundation model for cine cardiac MRI image analysis called CineMA. It is a masked autoencoder with a convolution-transformer architecture

that operates on both long-axis and short-axis images. The model was trained on millions of cine images from ~70K subjects in UK Biobank (UKB) using a mean square error loss evaluating its ability to reconstruct masked image patches. It is evaluated in UKB and in 8 additional datasets for a variety of fine-tuning tasks: (1) image segmentation (ventricle, myocardium and scar), (2) ejection fraction quantification (both segmentation-derived and directly regressed), (3) disease classification, (4) landmark localization and associated calculation of long-axis functional measurements and (5) regression of age and BMI and classification of sex and MRI vendor. The accuracy of the fine-tuned foundation model is typically compared with that of two types of models trained from scratch: a UNet or ResNet model as appropriate, or a CineMA model trained from scratch. The foundation model's performance outperforms these baselines or occasionally matches them. The authors also include an investigation into the association of long-axis functional measurements with diabetes, hypertension, cancer and survival in UKB.

Strengths:
- Foundation models are of high utility, and this one is trained on a large dataset and exhaustively validated for a large variety of fine-tuning tasks in many other datasets. I am convinced that it is likely to be an excellent starting point for model training by other researchers with high potential impact and immediate interest.
- The machine learning methods are sound.

Response: Thank you for recognising the potential utility in our work and the value of the exhaustive evaluations. Our primary objective was to establish a high-fidelity starting point for cardiac CMR research through rigorous validation across heterogeneous datasets. We are confident that the release of codebase and pre-trained/finetuned weights will lower the barrier to entry for the community, facilitating downstream applications in cardiac imaging and foundation model research.

Weaknesses:
- The baselines are incomplete. The authors cite three other foundation models that they claim have been insufficiently validated [17-19], including models from their own group. Is it not possible to compare to them? In addition, as you are operating on large public datasets/challenges, are there results from other groups that you can compare to?

Response: All these references come from different groups. For the work from Jacob et al. [18] the source code is not available. However, their recent version (published after our initial submission) has included comparisons to our models, where comparable performances were reported on both segmentation and disease classification tasks for SAX view.

Direct comparison with Zhang et al. [19] (ViTa), a study focusing on tabular metric prediction, is complicated by two aspects: ViTa jointly encodes CMR images with tabular patient-level factors (demographics, metabolic, lifestyle data) that were not used in CineMA pre-training, and it processes a fixed subset of six SAX slices rather than the

complete volume. Nonetheless, we have included their reported performance metrics for systemic diseases classifications on UKB dataset as reference benchmarks where possible (Supplementary Table 12), allowing readers to contextualise both models' results. We can see similar range of values, especially the same trend where diabetes classification is more accurate than hypertension from CMRs.

Table 12 Classification performance (ROC AUC) for systemic diseases and survival prediction on UK Biobank. CineMA was fine-tuned end-to-end using three random seeds. Performance from ViTa [9] was not evaluated on the same dataset, included for reference only. ROC AUC, area under the receiver operating characteristic curve.

Task	GLM	XGBoost	$\text{CineMA}^{\text{FineTune}}$	ViTa [9]
Hypertension	70.93	69.68	77.14	74.6
Cancer	55.35	52.75	65.50	
Diabetes	70.91	65.89	87.65	81.3
1-Year Survival	59.01	41.72	72.71	
2-Year Survival	59.33	51.58	68.25	
3-Year Survival	62.14	59.28	74.78	

For the work from Shad et al. [17], it has been compared with CineMA on Kaggle dataset, where their work reported a mean absolute error of 6.88%, while CineMA achieved ~4% error. Their source code was only available recently, after the time of submission. However, setting up their codebase proved non-trivial due to unclear integration points, compared to nnUNet. Given their model's reported performance on overlapping benchmarks and the inclusion of nnUNet as a direct baseline in our revision, we did not consider a separate comparison necessary.

Revision: We have now also included the competition results from literatures on ACDC, M&Ms, and M&Ms2 datasets as reference when possible with same metrics. The top methods were mostly nnUNet based with further task related data augmentation and other engineering.

- The authors claim that "nnUNet was not included in benchmarks due to its automated optimization pipeline. Fair comparison with such an AutoML optimization to foundation models remains an open research question". I find this unconvincing - readers will want to know if they should use your foundation model or train another model from scratch. Ideally, CineMA would outperform nnUNet as it is so often used by the field, and shown to often outperform alternative UNet setups.

Response: We agree with the practical importance of this comparison, although we note that it is crucial to maintain the distinction regarding its nature. nnUNet functions as an AutoML framework that dynamically reconfigures its architecture, preprocessing, and training hyperparameters for every specific dataset. In contrast, CineMA is a foundation model designed to provide robust, high-performance features through a standardised fine-tuning protocol. Because no equivalent automated hyperparameter optimisation pipeline currently exists for the fine-tuning of large-scale foundation models, the comparison inherently favours the AutoML approach on a per-task basis.

By including the comparisons on segmentation tasks, we demonstrate that CineMA achieves comparable performance to nnUNet on segmentation accuracy and better consistency across longitudinal studies even without the benefit of task-specific architectural search. Notably, when evaluated on entirely unseen external distributions (the Kaggle and Rescan datasets), CineMA demonstrates distributional resilience. Despite the reduction in training data, CineMA obtained comparable LVEF prediction accuracy to nnUNet while maintaining lower coefficient of variation. Consequently, CineMA proves more reliable for longitudinal clinical monitoring where consistency across heterogeneous acquisition environments is essential, highlighting the inherent strength of our pre-trained representations.

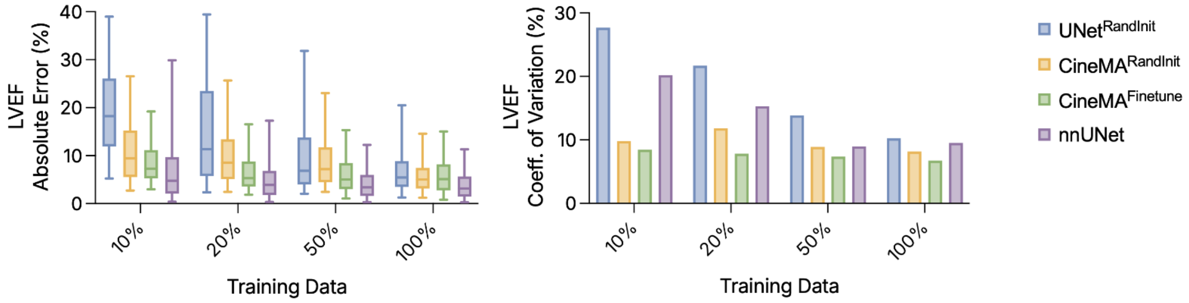

Revision: Following your suggestions and feedback from the community, we have also added a dedicated section to highlight how the proposed foundation models can be used, based on the task types and availabilities of data.

Practitioners should prioritise direct inference for short-axis segmentation using the M&Ms-2 fine-tuned model, which has the largest training set and demonstrates the best performance on unseen data distributions. Prediction stability can be further augmented through model ensembling across varying training seeds and datasets. In scenarios where local labels are accessible, CineMA serves as a robust alternative to nnUNet, particularly in low-data regimes where pre-training provides a regularisation effect. For disease detection and classification, the released weights enable immediate inference if the pathologies align with the ACDC or M&Ms cohorts, such as hypertrophic cardiomyopathy or myocardial infarction, whereas fine-tuning the CineMA backbone is recommended over standard ResNet architectures for novel conditions. For long-axis functional estimation, the provided weights allow for initial zero-shot validation on custom cohorts prior to adaptation. For downstream tasks not evaluated in this study, such as image registration or super-resolution, the pre-trained CineMA encoders function as optimised feature extractors; their latent embeddings capture complex cardiac geometry that can be effectively transferred to predict velocity fields or recover sub-pixel anatomical details. Finally, for population-level studies, CineMA models enable the high-throughput derivation of digital biomarkers, including cardiac functional indices and automated disease prevalence estimates. When integrated into prognostic models or risk stratification pipelines, these CineMA-derived metrics facilitate complex multi-modal association studies while providing critical image-based explicability for downstream clinical predictions.

- The authors claim that their model can perform prognostication, but in actuality the CineMA model is used to derive ejection fractions and long-axis functional measurements that are in turn used to train a Cox proportional hazards model. It would be much more interesting if the foundation model's representation itself was used to fine-tune a model for prognosis, as that would much better illustrate the utility of the learned representations for downstream tasks.

Response: We agree that leveraging the foundation model's latent representations directly offers a more rigorous validation of the learned features than relying on derived clinical metrics. Consequently, we performed end-to-end fine-tuning of CineMA on UK Biobank (UKB) data to predict hypertension, cancer, diabetes, and longitudinal survival (1, 2, and 3-year outcomes). As shown in Figure 4, these fine-tuned models achieved superior performance over baseline models trained strictly on derived functional metrics, reaching convergence within 11 epochs. Notably, this approach overcomes the significant barriers of training from scratch on the entire UKB dataset, where the high-dimensional input image with 50 time frames would make training computationally prohibitive and unstable. These results demonstrate that CineMA's pre-trained embeddings capture complex, prognostic signatures that transcend standard volumetric indices, thereby confirming the utility of the foundation model for downstream clinical tasks with high computational efficiency.

Revision: we fine-tuned CineMA and have updated figure 4, section 3.7 and added supplementary table 12.

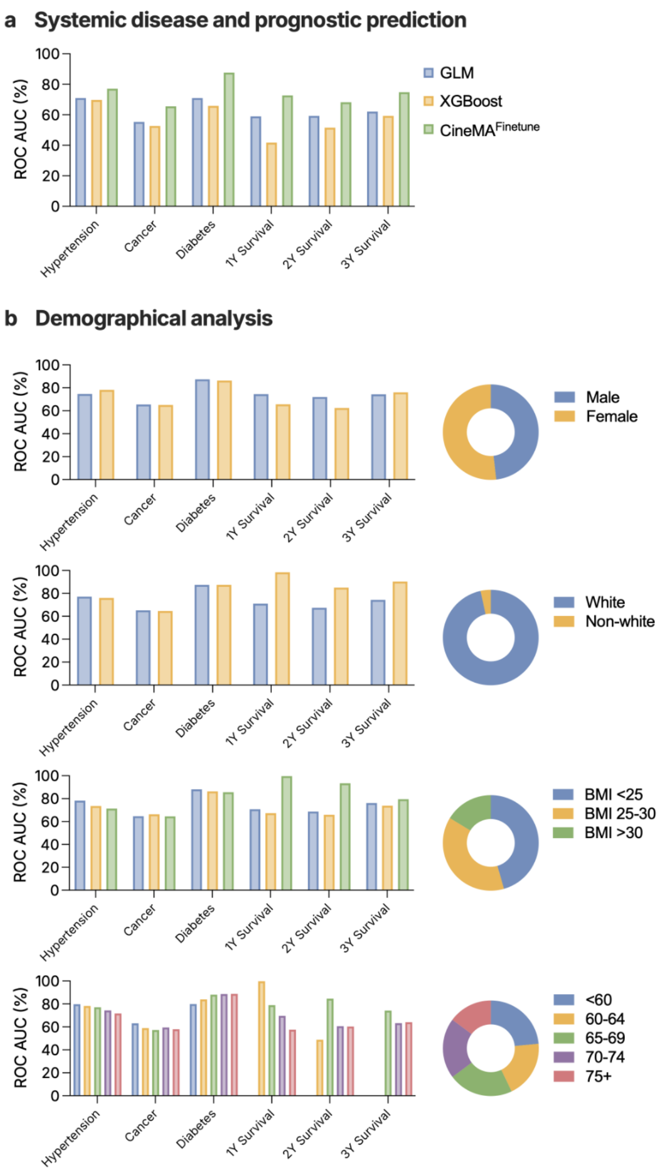

Table 12 Classification performance (ROC AUC) for systemic diseases and survival prediction on UK Biobank. CineMA was fine-tuned end-to-end using three random seeds. Performance from ViTa [9] was not evaluated on the same dataset, included for reference only. ROC AUC, area under the receiver operating characteristic curve.

Task	GLM	XGBoost	$\text{CineMA}^{\text{FineTune}}$	ViTa [9]
Hypertension	70.93	69.68	77.14	74.6
Cancer	55.35	52.75	65.50	
Diabetes	70.91	65.89	87.65	81.3
1-Year Survival	59.01	41.72	72.71	
2-Year Survival	59.33	51.58	68.25	
3-Year Survival	62.14	59.28	74.78	

Additional comments and questions:

- The statistical reporting in several tables is incomplete, as means are often reported without corresponding uncertainty measures (e.g., standard deviations)

Revision: We have added standard deviations in all tables.

- Is your foundation model applicable to other tasks, e.g., registration, cardiac motion analysis or super-resolution?

Response: The proposed foundation model provides a task-agnostic encoder for both SAX and LAX view, which can be integrated into various architectures. For image registration and motion analysis, the pre-trained encoder serves as a feature extractor for both fixed and moving frames; these latent embeddings can then be processed through a task-specific head to regress velocity or displacement fields. Myocardial strain parameters can subsequently be derived from the predicted displacement. Similarly, for super-resolution, the encoder extracts latent features from low-resolution inputs, which can then be mapped to a high-resolution space.

Revision: We have updated the discussion section.

For downstream tasks not evaluated in this study, such as image registration or super-resolution, the CineMA encoders can function as pre-trained feature extractors, as their latent embeddings capture complex cardiac geometry that can be effectively transferred to predict velocity fields or recover sub-pixel anatomical details [34].

- Please explain further why CineMA-FineTune is almost universally worse than the Unet/ResNet models.

Response: Finetuned CineMA has achieved comparable or better performance to Unet/ResNet in segmentation (Supplementary Table 3-7), classification (Supplementary Table 8, 9), regression (Supplementary Table 10), and landmark localisation (Supplementary Table 11). However, as mentioned in discussions, the non-finetuned CineMA has indeed a performance gap compared to convolutional networks. This performance gap between pure convolutional architectures and Transformer-based architectures was also observed in previous work such as Jacob et al. [18] and Zhang et al. [19]. We hypotheses that the strong inductive biases such as locality inherent to convolutional networks provide a structural prior, which can be learned through unsupervised pre-training as shown in fine-tuned performances [31].

Revision: This has been included in the discussion section.

While the proposed convolution–transformer architecture yielded strong performance when fine-tuned, it did not consistently match purely convolutional architectures when trained from random initialisation. This performance gap was also observed in previous work [18, 19, 32]. Future work is required to optimise the architectural design.

- Section 2.8, do you have evidence that a fair model would predict a positive cardiac function outcome with equal probability across ethnic groups? Doesn't this ignore differing outcomes by race? Demographic disparity analysis is also for white vs. non

white only. It would be interesting to also evaluate males vs. females, age groups, etc, but perhaps not necessary for this paper.

Response: Given the fine-tuned models added in revision, we focused our fairness evaluation on predictive parity, examining if the model maintains consistent AUC across demographic subsets. Following your suggestion, we expanded our analysis beyond the initial white vs. non-white binary to include gender, BMI, and age-based groups. As illustrated in Figure 4, the fine-tuned CineMA model demonstrates comparable performance across demographic groups when predicting systemic diseases. We also observed higher performance variance in survival predictions, we suspect this is potentially due to inherent class imbalances.

Revision: we have updated figure 4 for fairness comparisons.

- The paper is clear and well-written, and the authors do provide enough detail for a researcher to reproduce the work.

Response: Thank you for recognising the clarity and reproducibility of our work.

Detailed:

- Authors should modulate their claim to be the first cine CMR foundation model, as they cite others [18,19] that were previously developed.

We have modulated the claims.

- As many datasets are used, clarity would be improved if all figure and table captions included which dataset was being used.

We have specified the datasets used per sub-figure and tables.

- Oftentimes, numbers in the main text do not correspond exactly to number in the associated tables. I assume that the numbers in the main text are averages of several experiments from the tables? Please clarify. An example: section 2.5, "specificity (60.06% vs 31.55%) and sensitivity (90.20% vs. 86.84%)", where these numbers are not in the corresponding Table 5.

As there are multiple fine-tuned models trained on different datasets and evaluated on different datasets, for main figures, we calculate the aggregated performance across the combined test set from multiple datasets. We have now clarified the aggregation in main text and figure captions. The aggregated results are now also listed in corresponding tables.

- Section 2.5: It would be helpful to list the diagnostic labels in the ACDC, M&Ms and M&Ms2 datasets, and to also show the prevalence of these diseases in the datasets.

We have added the distribution of these diseases in Table 2.

- Section 2.6: be clear that GLS is never statistically significant

We have clarified in the section:

Overall, fine-tuned CineMA achieved comparable accuracy as baseline models without a statistically significant difference, indicating a feasible alternative in these functional assessment tasks.

- Please clarify the y-axis in Figure 4b - does 0% "difference in mean" mean that it matches the group not surviving even 1 year? Also, why is there an opposite trend for LVEF vs RVEF?

We have replaced the analyses using fine-tuned CineMA model performances.

- Section 4.1: Are the UKB images from instance 2, instance 3, or both?
We used both instance 2 and 3, we have clarified in the main text.
- Section 5: add citation for the Landmark dataset
We have added the citation.
- Table 1 caption: add acronym for LGE, T2 and bSSFP
We have added the acronyms.
- Table 5: CineMA-RandInit has very low specificity for ACDC SAX and M&Ms SAX?
This is the results we obtained, where we observed unstable training and the models were not able to learn the normal class. Given that using same code and training setting with ResNet, we obtained reasonable results, we consider this as a limitation in the tested architecture instead of a bug. We hypothesis that the underperforming is because all Transformer encoded image patches were averaged without gradual downsampling as in ResNet, this reduce the model's expression capacity. The better performance achieved by fine-tuned models further demonstrates the benefits of the pre-trained foundation models.

Typos:

- There are several figures and tables that are never cited in the main text (e.g., Figure 2a, Table 3, Table 9, and perhaps others).

We have fixed this.

- Page 2: "global systolic functon(e.g., LVEF) AND long-axis functon(e.g., MAPSE and GLS)
We have corrected it.
- I suspect the y-axis label in Fig 2c top-right "mean ejection fraction (%)" is a typo?
It should be ejection fraction mean absolute error. We have corrected it.
- Section 2.4: "mean errors of 3.72% for LVEF" but all errors in Figure 2c are >5%?
It was a typo and we have updated the text and added the corresponding data in supplementary tables.
- Figure 3c, order should be MAPSE, GL shortening for consistency
We have corrected it.
- Consider moving section 4.6 after section 4.7 and tables 10,11 after table 12, to match

the flow of the manuscript
We have moved the corresponding sections and tables.

Reviewer #3 (Remarks to the Author):

General Assessment
This manuscript presents CineMA, a foundation model for cine cardiac magnetic resonance imaging (CMR), trained on over 15 million images from nearly 75,000 subjects. The authors propose a multi-view convolution–transformer masked autoencoder capable of performing segmentation, diagnosis, and prognostication tasks across multiple independent datasets.
The paper is technically sound, comprehensive, and well-written, with a remarkable scale of pre-training and a robust validation framework. Its emphasis on generalisability, fairness, and open-source availability makes it a significant advance toward clinically applicable foundation models in cardiovascular imaging.
The authors also thoroughly test the foundation model across several downstream tasks in a fine-tuning setup using diverse datasets, demonstrating robustness and transferability.
However, several clinical contextual elements could be expanded or refined in the discussion to improve completeness and translational impact.

Major Comments
(None: the manuscript is excellent overall. The following are minor but important suggestions to enhance clarity and clinical perspective.)

We sincerely thank you for the positive assessment and for describing our manuscript as excellent. We appreciate the insightful suggestions provided to enhance the clinical perspective of the work and have addressed each of these points in the revised manuscript.

Minor Comments and Suggestions
1. Clinical Scope – Missing Common Disease Entities
While CineMA is convincingly validated on multiple datasets for segmentation and diagnosis, the discussion omits clinically common entities frequently encountered in CMR:
• Perimyocarditis / Myocarditis: Given the growing clinical importance of inflammatory cardiac diseases, a brief acknowledgment of their absence and potential integration in future datasets would be valuable.
• Valvular disease: Cine imaging is routinely used to assess valvular pathologies such as aortic or mitral regurgitation. Even if such data were unavailable, this could be mentioned as a potential extension for future work.

Response: We have extended discussions to include these conditions.

Furthermore, the evaluation datasets did not include myocarditis or other inflammatory cardiomyopathies, nor valvular pathologies such as aortic or mitral regurgitation, which

are commonly assessed using cine CMR in clinical practice. Future work should incorporate additional modalities and disease entities to broaden CineMA's clinical applicability, particularly for inflammatory conditions and valvular heart diseases.

2. Imaging Modalities – Missing T2-STIR Sequences
The authors note that CineMA was not trained on LGE and T1 mapping sequences, but T2-weighted STIR imaging, which is critical for oedema and inflammation detection, is also absent.
A brief discussion of this omission would be appropriate, clarifying that incorporating T2-STIR or T2 mapping could extend CineMA's clinical utility, especially for myocarditis and acute injury detection.

Response: T2 images were included in Myops2020 dataset (n=25), but the data is very limited. We have extended the introduction and discussions to include the importance of these modalities and suggest the potential next steps.

Cardiac magnetic resonance images (MRIs) encompasses multiple sequences, including cine imaging for functional assessment, late gadolinium enhancement (LGE) for scar detection, and T1/T2 mapping for tissue characterisation. Among these, cine imaging is the most widely acquired sequence and enables quantification of cardiac size (e.g. cavity volume), myocardial mass, global systolic function(e.g. left ventricle ejection fraction [LVEF]), and long-axis function (e.g. mitral annular plane systolic excursion [MAPSE], and global longitudinal shortening [GLS]).

In addition, we observed limited benefits of CineMA on LGE, T1, and T2 mapping images (Supplementary Table 13, Supplementary Table 14), which is expected given that these modalities were not included in pre-training. T2-weighted imaging, including T2-STIR sequences critical for detecting myocardial oedema in myocarditis and acute cardiac injury, was only available in the limited MyoPS2020 dataset (n=25). Furthermore, the evaluation datasets did not include myocarditis or other inflammatory cardiomyopathies, nor valvular pathologies such as aortic or mitral regurgitation, which are commonly assessed using cine CMR in clinical practice. Future work should incorporate additional modalities and disease entities to broaden CineMA's clinical applicability, particularly for inflammatory conditions and valvular heart diseases.

3. Inter-Reader Variability and Echocardiographic Comparison
The introduction correctly highlights the high inter- and intra-reader variability of manual CMR interpretation.
It would strengthen the discussion to note that echocardiography, which is often used alongside CMR, further contributes to this variability.
CineMA's foundation model approach could introduce a more objective framework for ejection fraction estimation and functional assessment.
Emphasizing this would underline CineMA's potential for clinical reliability and standardization in quantitative cardiac function evaluation.

Response: We have extended the introduction to include echocardiography and emphasize the potential of foundation models.

Revision: We have discussed the important to address inter- and intra-reader variability in introduction and result section 3.4.

This variability is compounded when CMR measurements are compared with echocardiography, which remains the first-line imaging modality in many settings. With the advancement of deep learning, automated image interpretation has become feasible, helping to alleviate clinician workload, standardise quantitative cardiac assessment and improve clinical workflow efficiency [6–13].

These results suggest that although nnUNet's dataset-specific tuning strategy improves performance on similar data distributions, it may be less robust to distribution shifts. In contrast, pre-training regularises CineMA's representation learning during fine-tuning, yielding better generalisation capacity, particularly in low-data settings. This consistency across heterogeneous acquisitions addresses a key clinical need: reducing the operator- and scanner-dependent variability that currently limits CMR interpretation, thereby supporting more standardised cardiac function assessment in multi-centre studies and longitudinal monitoring. Consequently, CineMA can be considered a viable alternative to nnUNet for clinical studies, reducing the manual annotation burden while preserving generalisation across populations.

4. Presentation of Systemic Disease Associations
The manuscript repeatedly highlights associations with systemic diseases (diabetes, hypertension, cancer) across multiple sections, not only in the discussion but also in the abstract and results.
While these findings are interesting, they should be presented with greater caution and positioned as secondary or exploratory analyses, rather than as central outcomes.
A clear statement that these represent correlations rather than causal relationships would improve scientific precision.
Reframing these results as supporting evidence for generalisability, rather than as primary validation findings, would yield a more balanced interpretation.

Response: We have replaced these analyses by fine-tuning CineMA models to predict directly systemic diseases and survivals (Figure 4). For fairness analyses, we compared model performance across demographic groups (sex, race, BMI, age). We have also acknowledged that these tasks are difficult and our results remain exploratory analyses to ensure a more balanced and scientifically precise interpretation of our results.

Revision: The new results are summarised in Section 3.7.

Following the improved classification of cardiovascular diseases, we conducted exploratory analyses to evaluate whether CineMA^{FineTune} representations could capture subtle

phenotypical signatures associated with systemic comorbidities (hyper-tension, cancer, and diabetes) and longitudinal survival (one-, two-, and three-year horizons). To preserve learned features and reduce training time, the encoder was frozen except for the last block, enabling convergence within 20 epochs. Despite the inherent difficulty of these tasks, subtle cardiac manifestations of systemic conditions and severe class imbalance in survival prediction, CineMA^{FineTune} achieved ROC AUC of 77.14% for hypertension, 65.50% for cancer, and 87.65% for diabetes (Supplementary Table 12). For survival prediction, ROC AUC ranged from 68.25% (2-year) to 74.78% (3-year). Across all tasks, CineMA^{FineTune} consistently outperformed GLM and XGBoost baselines trained on model-derived morphometric metrics, including ventricular volumes and ejection fraction from SAX views alongside ventricular length and function estimations from LAX views (Figure 4, Supplementary Fig. 3). This suggests that the latent representations encode prognostically relevant information beyond conventional morphometric parameters, demonstrating the potential of foundation models to support clinical risk stratification. Validation in external cohorts will be an important next step toward clinical translation.

Summary
This is an outstanding and forward-looking contribution to the field of cardiac imaging AI.
I recommend minor revisions focused on enhancing the clinical discussion by:
• acknowledging missing disease entities (myocarditis, valvular disease),
• mentioning non-utilised but clinically important sequences (T2-STIR),
• emphasizing CineMA’s role in mitigating inter-reader variability (including in echocardiography), and
• presenting systemic disease associations more cautiously as secondary exploratory findings.
These revisions will make the manuscript more clinically balanced, precise, and aligned with real-world cardiovascular imaging practice, without requiring additional experimentation.

We once again sincerely thank the reviewer for the encouraging assessment and for the constructive suggestions that have meaningfully strengthened the clinical perspective of our manuscript.

Dear Editor,

Thank you again for the opportunity to revise our manuscript, "Development and validation of a versatile foundation model for cine cardiac magnetic resonance image analysis." We are grateful for the reviewer's recommendation for acceptance and the editorial team's detailed formatting guidance. We have addressed the remaining comments. A point-by-point response to reviewer comments follows below. We have also addressed all editorial requests: section numbering and the abstract code availability sentence have been removed; an ethics statement has been added; source data for Figures 2–4 are compiled into Supplementary Data 1 with a note in the Data Availability section; and all supplementary files have been merged into a single PDF in numerical order.

Best regards,
Yunguan Fu

Reviewers' comments:
Reviewer #1 (Remarks to the Author):
Thank you for addressing my comments.

Reviewer #3 (Remarks to the Author):
The authors have addressed the concerns raised in my previous review in a clear and satisfactory manner. The revisions have improved the methodological transparency and clarity of the manuscript. I have no further comments.

We thank Reviewer #1 and Reviewer #3 for their positive assessment and confirmation that their concerns have been fully addressed.

Reviewer #2 (Remarks to the Author):

I would like to thank the authors for addressing my original concerns, and would wholeheartedly recommend this paper for acceptance.

The authors have created a foundation model using state-of-the-art methods and have performed a comprehensive assessment from a variety of angles that demonstrate its generalizability and where it is sometimes outperformed by other methods (e.g., nnUnet in the case of full training data).

We sincerely thank Reviewer #2 for the positive assessment and thoughtful recommendations, which have further strengthened the manuscript. We address each point below.

I have a few recommendations for the final manuscript:

- Section 3.4, "CineMAFineTune achieved comparable LVEF prediction accuracy to nnUNet while maintaining a low coefficient of variation (≤ 10.10%), whereas nnUNet’s variation increased to 20.20%".

Consider revising this sentence to clarify that this refers to the situation when 10% of training data is available.

We agree that clarifying the increased coefficient of variation of nnUNet was only when 10% of training data is available. We have revised the sentence as below:

[...] evaluation on the Kaggle and Rescan datasets demonstrates that $CineMA_{FineTune}$ achieved comparable LVEF prediction accuracy to nnUNet. Using only 10% of the available training data, $CineMA_{FineTune}$'s coefficient of variation increased modestly from 6.73% to 8.47%, whereas nnUNet's increased from 9.53% to 20.20%.

- Regarding the hyperparameter selection for the baseline and new models - you are using a fixed number of epochs and a fixed learning rate decay strategy for all tasks and all models. Did you consider using a strategy for learning rate decay and early stopping that is based on validation loss (e.g., keras EarlyStopping, ReduceLROnPlateau and ModelCheckpoint options)? You'd have to choose parameters for patience and learning rate reduction factor to share across all tasks, but this might better automatically adjust the training for each task compared to the fixed parameters you have now but it might be worth considering mentioning in the discussion or for your group's future work.

We used early stopping based on validation performance, stopping training if the task-specific validation metric did not improve for 5 consecutive evaluation rounds. The learning rate followed a linear warmup and cosine decay schedule. We have added an explicit mention of the learning rate schedule in the revised manuscript to improve clarity:

A linear warmup followed by cosine decay learning rate schedule was used, and early stopping was employed based on task-specific validation metrics.

We note that our cosine decay schedule and ReduceLROnPlateau represent alternative approaches to learning rate scheduling. Exploring adaptive strategies is a promising direction for future work. We further discussed this in discussion:

Without extensive hyper-parameter tuning per dataset, CineMA demonstrated competitive segmentation accuracy to nnUNet and more consistent estimates of ejection fraction, particularly in low-data scenarios and in external datasets with population shifts. Task-specific optimisation of hyperparameters, including the data augmentation strategy, training schedule, and prediction head architecture, may further close this gap and is left for future work.

- Supplemental Table 1: I don't think 'Dice loss + CE loss' was the loss for the heatmap regression?

The landmark localisation via heatmap approach uses Dice loss + CE loss. Concretely, each of the three landmarks is converted into a Gaussian heatmap, yielding a three-class soft segmentation mask. Because the heatmaps are treated as soft segmentation targets, Dice loss combined with cross-entropy loss is used as both loss functions support soft labels. We now name the two approaches for landmark

localisation as heatmap-based and coordinate-based, without using the word “regression” which may lead to confusion. We have clarified this in the revised manuscript:

For each image, a heatmap was generated per landmark using a Gaussian kernel with sigma = 3. The three landmark heatmaps form a three-class soft segmentation mask, and Dice loss combined with cross-entropy loss on this mask was used for training.

- In all tables, consider bolding the best results, to highlight the best model for readability

We have made the best results bold per metric.

- Results of the Wilcoxon signed-rank test are missing for Figure 2c and perhaps 3c (maybe these ones were all not significant).

We have added the statistical significance testing results in Figure 2c. For Figure 3c, none of the comparisons are significant. We have clarified this in the caption.

- I recommend calculating AUPRC for the results in Figure 4 or indeed anywhere you may have class imbalance (e.g., diabetes prediction).

We have computed AUPRC for all systemic disease and survival prediction tasks, where class imbalance is observed. Both AUROC and AUPRC are now reported in Figure 4 and Supp. Table 12. CineMA outperformed baselines on AUPRC across all systemic disease tasks. For survival prediction, AUPRC remained low across all models due to extreme class imbalance. CineMA achieved the highest AUPRC except for 3-year survival, where XGBoost scored higher. However, the precision-recall curves (Supp. Figure 3) confirm that beyond 10% recall, CineMA's precision exceeds baselines. The result section has been updated:

$\text{CineMA}_{FineTune}$ also achieved higher AUPRC than baselines for all systemic disease tasks (56.62% for hypertension, 34.10% for cancer, and 30.54% for diabetes). For survival prediction, AUPRC remained low across all models due to extreme class imbalance (positive rates of 0.10--1.19%). $\text{CineMA}_{FineTune}$ outperformed baselines across all tasks except 3-year survival AUPRC, where XGBoost achieved a higher score (6.35% vs 2.82%) despite a substantially lower AUROC (59.28% vs 74.78%). However, as shown in the precision-recall curves, XGBoost's precision falls below that of $\text{CineMA}_{FineTune}$ beyond 10% recall.

- For your new Figure 4 results predicting hypertension, cancer, and diabetes, are you predicting incident or prevalent disease? If incident, is it with respect to the imaging date and if so, how do you handle instance 2 vs. instance 3 images?

These are prevalent disease predictions. We have clarified the relevant terminologies and descriptions in the method:

We used UKB for predicting diabetes, cancer, hypertension, and survival status. Hypertension, cancer, and diabetes are prevalent disease predictions, as the labels reflect whether each condition had ever been diagnosed at the time of data retrieval, rather than incident events occurring after imaging. The labels of

hypertension and cancer were derived from data field 2966 (age high blood pressure diagnosed) and 40008 (age at cancer diagnosis), respectively. As UKB regularly updates the metadata, for individuals diagnosed with hypertension or cancer after the image acquisition, i.e. the age at diagnosis is larger than the age at acquisition, the labels were set to negative.

We have also added in discussion regarding incident disease prediction:

Future work may also evaluate outcome-prediction models on additional clinical endpoints and external cohorts to determine the added value of CineMA for improving prognostic models and risk stratification pipelines. Furthermore, the systemic disease predictions in this study reflect prevalent conditions; investigating incident disease prediction, where the model identifies individuals who will subsequently develop a condition, would be a clinically valuable extension.
